# Genomic Signatures of Microgeographic Adaptation in *Anopheles coluzzii* Across Urban, Rural, and Forested Environments in Gabon

**DOI:** 10.1111/mec.70349

**Published:** 2026-04-20

**Authors:** Josquin Daron, Lemonde Bouafou, Jacob A. Tennessen, Nil Rahola, Loic Talignani, Boris Makanga, Ousman Akone‐Ella, Marc F. Ngangue, Neil M. Longo Pendy, Christophe Paupy, Daniel E. Neafsey, Michael C. Fontaine, Diego Ayala

**Affiliations:** ^1^ MIVEGEC, Univ. Montpellier, CNRS, IRD Montpellier France; ^2^ CIRMF Franceville Gabon; ^3^ Department of Immunology and Infectious Diseases, Harvard T.H. Chan School of Public Health Boston Massachusetts USA; ^4^ Infectious Disease and Microbiome Program Broad Institute of MIT and Harvard Cambridge Massachusetts USA; ^5^ CENAREST Libreville Gabon; ^6^ ANPN Libreville Gabon; ^7^ Groningen Institute for Evolutionary Life Sciences (GELIFES) University of Groningen Groningen the Netherlands; ^8^ Medical Entomology Unit, Institut Pasteur de Madagascar Antananarivo Madagascar

**Keywords:** *Anopheles coluzzii*, local adaptation, malaria vector, selective sweeps, standing genetic variation, urban to forest adaptation

## Abstract

Species distributed across heterogeneous environments often evolve locally adapted populations, but understanding how these persist in the presence of homogenizing gene flow remains puzzling. In Gabon, *Anopheles coluzzii,* a major African malaria mosquito, is found in various ecological settings, including urban areas, remote rural villages, and forested environments away from any human presence. This study investigates the genomic signatures of local adaptation in populations from distinct environments including the urban area of Libreville, and two proximate sites 10 km apart in the La Lopé National Park (LLP), a village and its sylvatic neighbourhood. Whole genome re‐sequencing of 96 mosquitoes unveiled 5.9 million high‐quality single nucleotide polymorphisms. Coalescent‐based demographic analyses suggest an *∼*12,000‐year‐old divergence between Libreville and La Lopé populations, followed by a secondary contact (*∼*4000 ybp) resulting in asymmetric effective gene flow. The urban population displayed reduced effective size, evidence of inbreeding, and strong selection pressures likely associated to insecticides or pollution present in urban settings, as suggested by the hard selective sweeps detected in genes involved in detoxification and insecticide resistance. In contrast, the two geographically proximate LLP populations showed larger effective sizes, and distinctive selective signals, notably soft‐selective sweeps on the standing genetic variation. Although presumably neutral loci failed to discriminate between LLP populations, our findings support that microgeographic adaptation can swiftly emerge through selection on standing genetic variation despite gene flow. This study contributes to the growing understanding of evolution of populations in heterogeneous environments amid ongoing gene flow and how major malaria mosquitoes adapt to humans and its environment.

## 1 Introduction

The genetic mechanisms by which natural populations can adapt to heterogeneous habitats has been a fundamental question in ecology and evolution. Local adaptation is responsible for the acquisition of traits providing a selective advantage under specific environmental conditions, regardless of the fitness consequences in other habitats (Tiffin and Ross‐Ibarra 2014). At the molecular level, local adaptation can be associated with *de novo* mutation where one haplotype carrying the advantageous allele can quickly reach fixation through “hard” selective sweeps. However, this process may be relatively slow to evolve. Instead, local adaptation can emerge from recycling preexisting standing genetic variants (SGV) or by multiple independent, but functionally equivalent, mutations at a genes through “soft” selective sweeps (Garud 2023). Because hard selective sweeps are generally easier to detect using outlier approaches (Smith and Haigh 1974), the vast majority of empirical work has been centered upon a “hard‐sweep” model of adaptation. However, a growing body of literature suggests that soft sweeps might be a more frequent mode of adaptation in many natural populations (Garud et al. 2015; Hermisson and Pennings 2005; Pritchard et al. 2010; Schrider and Kern 2017; Small et al. 2023). More importantly, both selection modes (hard and soft) are fully compatible and complementary, and sometime hard to discriminate (Harris et al. 2018; Johri et al. 2022). Consequently, understanding the relative interplay between new mutations and SGV for the adaption process is a key challenge to understand the ability of species to evolve into changing environments (Visser 2008).

The African malaria mosquito *Anopheles coluzzii* exhibits a remarkable ecological ability to proliferate in a broad range of habitats as diverse as humid rain‐forest, highland, and dry savannah (Simard et al. 2009; Tene Fossog et al. 2015). The ecological success of *An. coluzzii* is directly rooted in the tremendous genetic and chromosomal polymorphisms and in its close association with human settings (Ag1000G consortium 2017, 2020; Ayala et al. 2014, 2017; Fontaine et al. 2015). Humans provide blood meals, as well as shelters, and breeding sites to *An. coluzzii* (White et al. 2011). This degree of specialization on humans has drastically impacted the recent evolution of *An. coluzzii* (Coluzzi et al. 1979). First, the adaptation to newly modified landscape by the advent of agriculture 5000–10,000 years ago (yra) and human density impacted its evolution by greatly increasing their population sizes (The Ag1000G Consortium 2017, 2020). Second, the massive use of insecticides for malaria control in the second half of the last century selected resistant variants within natural populations of this mosquito by genetic changes at targeted sites in the genome (i.e., *de novo* mutations; Liu 2015; Ranson and Lissenden 2016). Third, the rapid and recent development of large cities in Africa led to strong selection of genetic and physiological factors (e.g., osmoregulation) (Tene Fossog et al. 2015) to tolerate urban pollution through detoxification (Kamdem et al. 2017). There is growing evidence for adaptation to various anthropogenic pressures, including broad‐spectrum insecticide resistance and successful breeding in polluted, man‐made habitats—traits observed in both *An. coluzzii* (Ranson and Lissenden 2016) and *An. stephensi* (Enayati et al. 2020). Notably, resistance to insecticides in urban settings has been widely reported in *An. gambiae* and *An. coluzzii*, and is associated with a rapid rise in knockdown‐resistance (kdr) haplotypes, similar to patterns seen in *Aedes* species (Baltzegar et al. 2021).

Motivated by the profound implications for human health, malaria research has intentionally narrowed its focus to anthropogenic settings, neglecting less anthropized and wild areas (Ayala et al. 2009; Kyalo et al. 2017). In the last few years, several studies incidentally reported the presence of species within the *An. gambiae* complex in wild conditions away from any kind of permanent human presence in Gabon, South Africa or Madagascar (Munhenga et al. 2014; Paupy et al. 2013; Zohdy et al. 2016), but also in well studied areas such as in Burkina‐Faso (Tennessen et al. 2021). These findings challenge the assumption that these mosquitoes are strictly bound to anthropogenic habitats. For example, the discovery of a previously unknown species within the *An. gambiae* complex, *An. fontenillei*, but present away from anthropic settings, underscores our incomplete understanding of biodiversity in *Anopheles* (Barrón et al. 2019). Such observations also raise questions about the evolutionary mechanisms behind these mosquitoes' ability to adapt locally in the absence of human selection pressures, revealing a remarkable capacity to thrive across diverse eco‐anthropogenic conditions. Therefore, wild areas, such as National Parks or protected areas, provide a compelling opportunity to investigate the origin and evolution of the main malaria mosquitoes in Africa and possibly also about factors underlying their vectorial capacity, such as in the arbovirus vectors (Rose et al. 2020).

**FIGURE 1:**
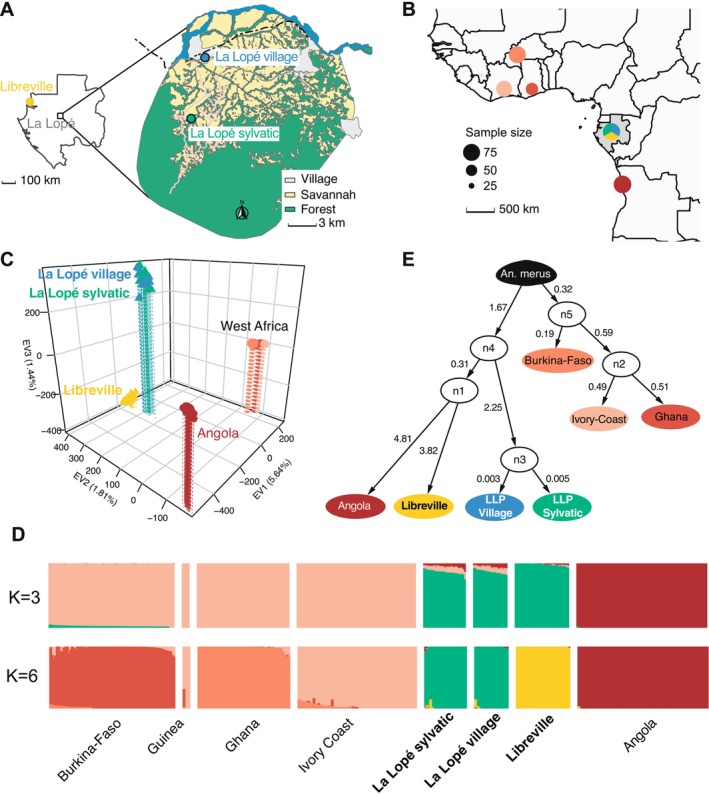
Geographic sampling and population structure and admixture of the three populations of *Anopheles coluzzii* in Gabon, in perspective with those of the Ag1000G consortium project. (A) Sampling locations of the three focal populations in Gabon. A total of 96 *An. coluzzii* mosquitoes were collected in Libreville as larvae, or as adults in the village and sylvatic area of the La Lopé National Park (see Table S1 for details). The geographic map of the National Park of La Lopé highlights the contrasted habitats that coexist in the park, with stable and permanent settlement of mosquitos observed in the village and the sylvatic areas. The base map was produced by digitizing the Gabonese land use freely available from the *Agence Nationale d'Étude et d'Observation Spatiale du Gabon* (http://ageos.ga). (B) Geographic locations of the 283 *An. coluzzii* mosquitoes analysed for the population structure at the scale of West Africa. Colours of regional groups are consistent throughout the study. The size of sampling point is proportional to the sample size. (C) Population genetic structure captured by the top three principal components of the PCA (see also the scree‐plot on Figure S2A). (D) *ADMIXTURE* analyses of the *An. coluzzii* populations. Each bar shows the genetic ancestry proportions for each individual to each genetic cluster tested and indicated by the *K* value on the left. The best fitting model according to the cross‐validation error rates was observed at *K* = 3 (Figure S5B), but finer sub‐structuration was visible until *K* = 6. See Figure S3 for the different solution from *K* = 2 to 9, and the associated cross‐validation (CV) error‐rates. (E) Most likely population graph topology recovered with a posterior probability of 95.04% using *AdmixtureBayes* (Nielsen et al. 2023). The population graph was rooted using *An. merus* used as outgroup species. The graph is composed of nodes (supported with posterior probabilities higher than 0.98) that are not the product of an admixture event (white circles). The numbers on the branch connecting populations capture the amount of genetic drift between populations. Branch lengths are grossly scaled by the amount of genetic drift accumulated along each branch of the tree.

Here, we explored the evolutionary forces impacting whole genome variation of *An. coluzzii*'s populations along three contrasted environments in Gabon, each characterized by a distinct level of human presence. By comparing populations from the forested (sylvatic) and rural areas of the La Lopé National Park (LLP), with an urban population from Libreville (LBV) and extending our analysis to a broader scale across Africa, we investigated the evolutionary processes by which these mosquitoes adapted to each environment. Given the permanent and stable occurrence of *An. coluzzii* not only in the rural villages of LLP, but also in the proximate forested areas (Ayala et al. 2019; Barrón et al. 2019; Paupy et al. 2013), we hypothesized that several genetic determinants may be involved in local adaptations to these environments (Figure 1A). To address these questions, we sequenced the whole genome of 96 mosquitoes sampled in Gabon. We investigated the population genetic variation, its spatial structure, and the interplay between evolutionary forces—genetic drift, migration and selection—shaping the population genetic variation in environments with varying degrees of human presence and types of habitats (city, remote rural village, and forest). We identified contrasted demo‐genetic dynamics between the urban LBV population and the populations from LLP. On one side, the urban population exhibited clear evidence of strong selective pressure marked by hard selective sweeps associated with adaptation to polluted waters and insecticide, typical of a highly populated and anthropized environment. On the other side, the populations from the remote LLP exhibited larger effective sizes and more diffuse genetic evidence of selection on SGV. Consistent with their close proximity (~10 km) in LPP, the rural and sylvatic populations shared the same genetic ancestry. Yet we identified several highly localized genomic regions showing signatures of positive selection, suggesting that SGV in this species supports local adaptation to contrasting selective pressures across these environments.

## 2 Material and Methods

### 2.1 Experimental Design

Field mosquitoes were collected at the National Park of La Lopé, the village of La Lopé, and the city of Libreville between September 2015 and November 2017 (Figure 1A and Table S1), according to the research permits required by the Gabonese government (AR0015/15/MESRS/CENAREST/CG/CST/CSAR) and the National Parks Agency (AE15011/PR/ANPN/SE/CS/AEPN). Based on previous studies (Mourou et al. 2010), we adapted the collection methods to each setting in order to improve efficiency. In the sylvatic and rural habitats of La Lopé National Park, adult mosquitoes were sampled using Human Landing Catches (with the approval of the National Ethical Committee from Gabon PROT N° 0031/2014/SG/CNE) and BG traps (Biogents). Samples from Libreville city were collected by larvae dipping. A total of 96 mosquito samples were used in this study (32 from each site; Table S1).

### 2.2 DNA Extraction, Genomic Library Preparation, Whole Genome Sequencing

Total genomic DNA was extracted using DNeasy Blood & Tissue Kit (Qiagen) following the manufacturer's instructions. DNA quality and concentration were estimated via PicoGreen (Promega). Genome library preparations took place at the Broad Institute using a Nextera XT Library Preparation Kit (Illumina). The 96 libraries were barcoded, multiplexed, and sequenced on five sequencing lanes of an Illumina HiSeq X instrument using a 300‐cycles run format (2 × 150 bp paired‐end reads).

### 2.3 Bioinformatic Data Processing

To ensure compatibility of our data with those from the Ag1000G consortium (2017, 2020), we followed the same bioinformatics protocols for SNP calling. Briefly, short reads were mapped to the *An. gambiae AgamP4* PEST reference assembly (Holt et al. 2002; Sharakhova et al. 2007) using *bwa‐mem* version 0.7.17 (Li and Durbin 2009) with default parameters. Individuals with an average genome coverage depth lower than 14× (*n* = 9) were excluded from downstream analyses (Figure S2). After removing PCR duplicates with Picard toolkit (2019) and performing INDEL realignment with GATK's *IndelRealigner* v.3.7 (McKenna et al. 2010), SNPs were called using GATK *Unified Genotyper* v.3.7. Low‐quality SNP calls were filtered by removing variants that failed any of the following filters: QD < 5, FS > 60 and ReadPosRankSum < −8. We also retained only variants located within the 63% of the genome previously classified as accessible in the Ag1000G consortium (2017, 2020). Variants were then annotated using *snpEFF* v.4.3 (Cingolani 2022) with default parameters. Lastly, we generated a final high‐quality SNP dataset using *vcftools* v.1.16 (Danecek et al. 2011) considering only biallelic SNPs, with genotypes with a genotype quality (GQ) higher than 20, discarding any variants with a missingness rate over 5% (*l‐miss* < 5%) and individuals (*n* = 9) with a missingness rate over 10% (*i‐miss* < 10%).

Molecular sexing of each sample was conducted by estimating the normalized coverage ratio of the mean sequencing depth for each chromosome over the coverage of the whole genome. This allowed a molecular sexing for each individual, which was unknown for the larvae from Libreville (Figure S2B). The degree of relatedness among individuals (or kinship coefficient) was estimated using *plink* v.1.9 (‐‐genome; Figure S3) (Purcell et al. 2007). Haplotype estimation, also known as statistical phasing, was performed using *SHAPEIT2* version v2.r904 (Delaneau et al. 2008) following the parameters used in the Ag1000G Phase 2 (Ag1000G Consortium 2020), including an effective population size (*N*e) of 1,000,000, default parameters for the MCMC settings, and a window size of 2 Mb. The reference haplotype panel and recombination maps from the Ag1000G Phase‐2 (Ag1000G Consortium 2020) (ftp://ngs.sanger.ac.uk/production/ag1000g/phase2/AR1/haplotypes/) were also used to guide the phasing in SHAPEIT2. Estimation of the ancestral versus derived allelic states of the SNPs (a.k.a. SNP polarization) was determined using an outgroup species, following the Ag1000G consortium (2017, 2020). Specifically, we polarized the SNP dataset using the consensus alleles defined from 10 *An. merus* from (Fontaine et al. 2015). Polarized and phased datasets were composed respectively of a total of 5,859,776 and 2,982,164 SNPs for the whole genome (see Figure S1 for a detailed workflow).

### 2.4 Population Genetic Structure

To explore population structure in a larger continent‐wide context, we merged our Gabonese SNP dataset with the published phase‐2 data from the Ag1000G project, considering only *An. coluzzii* species which include populations from Angola, Ivory Coast, Ghana, Guinea, and Burkina‐Faso (Ag1000G Consortium 2020). Joint analyses between samples from Gabon and *An. coluzzii* samples from the Ag1000G phase‐2 were performed by merging both VCF and keeping only SNPs at the intersection of both datasets. Following the Ag1000G Consortium methodologies, we investigated population genetic structure considering only the biallelic SNPs of the euchromatic freely recombining regions of chromosome 3 (regions 3R: 1–37 Mb and 3 L: 15–41 Mb), avoiding the peri‐centromeric regions, and also avoiding well‐known inversions on chromosome 2, heterochromatic regions, and the X chromosome. We removed also SNPs in linkage disequilibrium, excluding SNPs above an *r* ^*2*^ threshold of 0.01 in moving windows of 500 SNPs with a step size of 250 SNPs via *scikit‐allel* version 1.3.3 (Miles and Harding 2016), following recommendations of the Ag1000G Consortium. A minor allele frequency (MAF) of > 1% was also applied. This procedure provides a genomic sampling of presumably nearly neutral SNPs data that can adequately represent the population genetic structure, while minimizing potential selective effects, as previously shown in the Ag1000G consortium (2017, 2020).

We first visualized population genetic structure using a PCA on the 1,003,463 unlinked SNPs using *scikit‐allel*, considering genomic data from the Gabonese mosquito samples alone and combined with the data from the Ag1000G consortium. Then, we estimated individual genetic ancestry proportions using the program *ADMIXTURE* v.1.3.0 (Alexander et al. 2009), testing various numbers of clusters (*K*) ranging from 2 to 10. *ADMIXTURE* was run for each *K*‐value a 100‐time, in the form of 10 replicate × 10 datasets, with each dataset composed of a random sampling of 100,000 variants from the total number of unlinked SNPs dataset. The most likely number of ancestral populations (*K*) was determined using the CV error rate (Alexander et al. 2009). Although the lowest CV error rate was obtained for *ADMIXTURE* models with *K* = 3 ancestral populations, we found that further population sub‐divisions was clearly recovered in analyses allowing up to *K* = 6. These had clear support from other analyses (PCA and pairwise average *F*_ST_) and also from the previous study by the Ag1000G consortium (2020). From the 100 ADMIXTURE runs for each *K*, we used *CLUMPAK* version 1.1 with default settings to compare and produce major and minor clustering solutions. In parallel, average *F*_ST_ values were computed between all pairs of 8 populations, using the Hudson's *F*_ST_ estimator (Hudson et al. 1992) with standard error for each average computed using a block‐jackknife procedure in *scikit‐allel*. *P*‐values were estimated from the z‐score following the Ag1000G consortium (2017, 2020).

To further explore the genetic relationship among populations, we performed an admixture graph analysis using *AdmixtureBayes* (Nielsen et al. 2023). Graphs were estimated using the LD‐pruned and polarized dataset of which individuals from Guinea (from the Ag1000G phase‐2 dataset) were excluded due to their small sample size (*n* = 5). We ran three independent MCMC chains each consisting of 22,500,000 steps (option ‐*n* = 450,000), discarding the first 50% as burn‐in. All other parameters were left as default. Finally, to test whether rural and sylvatic LLP samples formed a single panmictic population, we compared the observed versus permuted joint site frequency spectrum (jSFS) using *δaδi* version 1.6.3 (Gutenkunst et al. 2009). One thousand permuted jSFS was created using *δaδi* build‐in function “scramble_pop_ids” from the dadi.Spectrum_mod which generates an average spectrum expected overall permutation of the individuals in the dataset. Departure of the observed jSFS from the permuted jSFS, under the null hypothesis of no population differentiation, were tested using a Chi‐2 test.

### 2.5 Genetic Diversity

We quantified the level of genetic diversity in each population by computing several descriptive statistics from chromosome arms 3L and 3R excluding the pericentromeric regions with *scikit‐allel*. Nucleotide diversity (π) and Tajima's *D* were calculated in 10‐kb non‐overlapping windows. Runs of homozygosity (ROH) were defined as contiguous regions of an individual's genome where only homozygous blocks were identified through a HMM function implemented in *scikit‐allel* as described in the Ag1000G Consortium (2017). Following the Ag1000G Consortium, all the autosomes were used for this analysis. Tracts of identity‐by‐descent (IBD) between all pairs of individuals within each of the 8 populations were inferred by *IBDseq* version r1206 (Browning and Browning 2013) with default parameters. Folded SFS (*a.k.a*. the minor allele frequency spectrum or MAF) was computed using allele counts using *scikit‐allel*. To facilitate comparison with theoretical SFS for a population with constant size (expected to have the constant scaled frequency for all values of *k*), we scaled each folded SFS by a factor (*k* * (*n*−*k*)/*n*) where *k* is the minor allele count and n is the number of chromosomes following the Ag1000G Consortium (2017). LD decay was computed by calculating the genotype correlation coefficient *r* ^*2*^ (Rogers and Huff 2009) for randomly sampled pairs of SNPs at distances raging from 10 to 10^7^ bp using *scikit‐allel*.

### 2.6 Demographic History

We estimated the long‐term demographic history of each population from Gabon using *Stairway Plot* 2 (Liu and Fu 2020). This approach estimates within population *Ne* variation back to the time of the most recent common ancestor (TMRCA) based on the unfolded SFS. The unfolded SFS was generated for each population using *scikit‐allel* from the peri‐centromeric euchromatic regions of chromosome 3 of the polarized SNP dataset (Ag1000G Consortium 2017). To translate *Stairway plot* estimates of *Ne* and time into natural units (i.e., individuals and years respectively), we assumed a generation time of 11 per year and a mutation rate of 3.5 × 10^−9^ per bp and per generation following the Ag1000G Consortium (2017).

We used *δaδi* to infer the best fitting demographic model of population isolation, possibly with migration, between populations from LBV and LLP village using the polarized SNPs dataset on chromosome 3, without any LD‐pruning nor any MAF filtering. We considered a total of eight alternative nested models of historical divergence, which were built on four basic population isolation models: (i) a model of Strict Isolation without gene flow (SI), (ii) a model of Isolation with continuous Migration (IM), (iii) a model of divergence with initial migration or Ancient Migration (AM), and (iv) a model of Secondary Contact (SC). These models were extended to integrate temporal variation in effective population size (G), enabling exponential growth or contraction, as previously suggested (the Ag1000G Consortium 2017). *Ne* variation implemented in *δaδi* models are thus necessarily simpler than in *Stairway Plot* 2 which split time to the TMRCA in as many chunks as needed for the likelihood optimization. In *δaδi*, the parameters number are kept low enough to enable convergence. The differences in demographic models between both approaches are thus not expected to match precisely, although they usually follow similar trends as previously noticed (the Ag1000G Consortium 2017). For each population, the SFS used as input for *δaδi* was computed for a number of individuals projected onto the smallest population sample size (*N* = 20 diploid samples for LLP village). A joined SFS (jSFS) between the pair of populations was generated for the sites in the genome that did not contain any missing data. The 8 models were fitted independently using successively a hot and a cold simulated annealing procedure followed by “BFGS” optimization (Tine et al. 2014). We set the grid points to {*n*, *n* + 10, *n* + 20}, where “*n*” is the number of haploid chromosomes (*n* = 40). Model parameter bounds for *Ne* scalars (*θ, nu1, nu2*) were *N* ∈ (0.01, 100), for the population exponential growth lambda parameter *b* ∈ (0.01, 100), for the time were *T* ∈ (0, 10), for migration were *m* **∈** (0, 50), and for the SNP orientation (polarization) uncertainty *O* ∈ (0.01, 0.99). We ran 100 independent optimizations for each model to check for convergence and retained the best one. Comparisons among models were based on the Akaike information criterion (AIC) (Table S2). We use the framework developed by Rougeux et al. (2017) to address over‐parametrization issue and to penalize models containing more parameters. We used a conservative threshold to retain models with ΔAIC < 10. Model parameters were converted into natural units as follows: ancestral effective population size (*Ne*) was calculated by *Ne* = *θ*/(4.*μ*.*l*), where *θ* is the scaled population mutation rate (*θ* = 4.*Ne*.*μ*.*l*), *μ* is the mutation rate per site and per generation (*μ* = 3.5 × 10^−9^), and *l* the length of the analysed sequence (*l* = 39,359,290). The effective sizes of populations 1 and 2 are given in units of *Ne_1_ = nu_1_ × N_ref_* and *Ne_2_ = nu_2_ × N_ref_*, where *nu_1_* and *nu_2_* are the population size relative to the size *N_ref_* of the ancestral population. Estimation of times in units of 2*N_ref_* generations (*T_s_* and *T_sc_*) were converted into years assuming a generation time of 0.09 years (equal to 11 generations per year) (Ag1000G Consortium 2017). Estimated migration rates (*m_12_* and *m_21_*) were divided by 2*N_ref_* to obtain the proportion of migrants received by each population every generation. The number of migrants per generation were obtained by *Ne_1_* × *m_12_* and *Ne_2_* × *m_21_*. To estimate parameter uncertainty, we used the Godambe information matrix method from *δaδi*. Bootstrapping was used to generate 1000 bootstrapped data sets to estimate the 95% confidence intervals (CIs) using the standard error of maximum likelihood estimates (se) (Table S2).

### 2.7 Identification of Selection Signatures

To identify candidate genes and genomic regions impacted by selection histories that varied geographically across sylvatic, rural, and urban areas, we first compared allele frequencies, haplotype diversity and homozygosity between sampling sites at the whole genome level. Genome scans of between‐population statistics were computed using *scikit‐allel* to report the Hudson's *F*_ST_ estimator statistic computed in blocks of 1000 SNPs along the genome. To detect long stretches of homozygosity in a given population relative to another population that could result from selective processes such as hard and soft selective sweeps (Sabeti et al. 2007; Garud et al. 2015; Garud and Rosenberg 2015), we first estimated the *H_12_* haplotype diversity statistics within each population using *scikit‐allel*. Comparisons between populations of longer stretches of homozygosity in one population relative to another were conducted with the XP‐EHH statistics using the *R* package *rehh* version 3.2.2 (Gautier et al. 2017) using the maxgap = 20 kb option to limit the extension of a haplotype through a gap of 20 kb. *P*‐value associated to each SNP was adjusted for the false discovery rate (FDR) with a threshold of 0.05. Candidate regions were identified using the R function *calc_candidate_regions* from the *rehh* package with a minimum number of significant SNPs in the region equal to 3 (min_n_extr_mrk = 3) and a windows size of 10 kb (window_size = 1e4). All genes spanning the candidate regions were reported in the Table S3.

We complemented the genome scan analyses of descriptive statistics with a model‐based genome scan that account for within population demographic changes (as estimated using *Stairway* *plot* 2) and key summary statistics, such as those we used above and others, using a deep learning approach relying on the convoluted neural network (CNN) implemented in *diploS/HIC* (Kern and Schrider 2018). This approach classifies genomic windows into five categories of selective sweep: (1) hard sweep, (2) linked‐hard sweep, (3) soft sweep, (4) linked‐soft sweep, or (5) neutral. Using the coalescent based simulator of *discoal* (Kern and Schrider 2016) informed by *Ne* variations from *Stairway Plot*, we first generated simulations of 110 kb genomic regions under the different categories of selection tested. These genomic regions were then further split into 11 sub‐regions of 10 kb to allow the CNN classifier to capture the genomic properties of the windows neighbouring the central focal window, based on a feature vector of 12 summary statistics previously selected for their ability to discriminate among the five selection categories tested (Kern and Schrider 2018). These statistics include *π* (Tajima 1983); 𝜃_w_ (Watterson 1975); Tajima's *D* (Tajima 1989); the variance, skew, and kurtosis of *g*_*kl*_ (the genotype distance between two individuals *k* and *l*); the number of multi‐locus genotypes, *J*_*1*_; *J*_*12*_; *J*_*2*_/*J*_*1*_ (equivalent to the *H*_*1*_, *H*_*12*_ and *H*_*2*_/*H*_*1*_ for phased data in Garud et al. 2015), the *Z*_ns_ statistics Kelly (1997), and the maximum value of ω Kim and Nielsen (2004). These statistics are then normalized across the 11 sub‐windows to capture the relative shape of each statistic across the larger region for each simulation under the various sweep model tested (See Kern and Schrider (2018) for further details). The simulations used as training and test datasets were produced using the same properties and demographic histories as observed in each of our three Gabonese populations under the five different types of selection scenarios. The demographic history generated by *Stairway Plot 2* for each population was used to generate simulated datasets as realistic as possible to our populations, following Xue et al. (2021), the Ag1000G consortium (2017), and A. Kern personal recommendations. A total of 2000 training genomic regions and 1000 testing regions with a single sweep were generated for each population, using as simulation parameters a per‐site and per generation mutation rate of 3.5 × 10^−9^ and 11 generations per year (The Ag1000G Consortium 2017). Once simulated, we first investigated the goodness‐of‐fit of the simulations to our observed data, and the performance and accuracy of the CNN classifier to discriminate among each of the models considered. These are critical steps to determine whether the model is well‐trained and to assess decisions that could be made from poorly fitting models (A. Kern, pers. comm.). We assessed the goodness‐of‐fit between the simulated and empirical data by visualizing the distribution of the observed values for each of the 12 descriptive statistics used by *diploS/HIC* compared to the simulated distributions obtained for each type of selection (Figure S10). The performance and accuracy of the CNN classifier to discriminate each model of selection under the simulated demography were estimated using the confusion matrix (Figure S11). In order to assess the convergence of the results, we generated 10 different coalescent simulated datasets for each population and each dataset was used to train 10 times the CNN classifier, resulting in a total of 100 runs. In order to compare and interpret the convergence between the predictions of each run, we first filtered out selective sweep having a low probability of being neutral (*p* > 0.01) and considered only sweep observed in at least 50% of our 100 replicated runs.

## 3 Results

### 3.1 Genetic Variation Across Sylvatic, Rural, and Urban Populations in Gabon

After read cleaning, mapping, SNP calling, and individual quality filtering, we retained a total of 87 individuals (out of the 96, Figure 1A) with an average sequencing depth coverage of 53.1 ± 25.01 × (Figures S1, S2, and Table S1). Nine additional individuals were removed because they exhibited a missingness rate over 10% (after genotype quality filtering) as well as one extra individual due to a high degree of relatedness (Figure S3 and Table S1). The final dataset thus included 77 individuals, sequenced at an averaged (median) ± SD [min—max] depth coverage of 57.1 (52.4) ± 23.2 [23.1–141.9], including 61 females and 16 males (Table S1 and Figure S2B). A total of 5.9 million high‐quality SNPs were obtained after data quality check and filtering (Figure S1). The SNP density along the genome included 1 SNP every 25 base‐pairs (bp) on average of the accessible compartment of the genome, with 86% of the SNPs being shared among samples from the three geographic sites: the urban area of Libreville city (LBV) and the two locations in the La Lopé National Park including a remote rural village area (LPV) and a sylvatic area 10 km to the South (LPS; Figures 1A and S2C). This indicates a high level of shared standing genetic variation (SGV).

### 3.2 Genetic Differentiation Between Libreville and La Lopé, Contrasting With Homogeneous Genetic Profiles at Presumably Nearly Neutral Loci Between La Lopé Rural and Sylvatic

We investigated the genetic structure of the Gabonese samples alone, and also in the context of the continent‐wide population structure of *An. coluzzii* (Figure 1B). For that purpose, we combined our data set with those from *An. coluzzii* of the Ag1000G consortium project (2020) (Figure 1B), focusing on the freely recombining regions of the chromosome 3, thus far from any low recombining regions, inversions, and heterochromatic regions, and using only unlinked SNPs. These SNPs are considered a good representation of nearly neutral genetic variation informative on the population genetic structure of the species, following the Ag1000G Consortium (2017, 2020). Focusing only on the Gabonese samples, the principal component analysis (PCA) showed that only the first PC axis was meaningful and revealed that the urban mosquitoes from Libreville (LBV) grouped tightly together and apart from those of the La Lopé National Park (Figure S4A). Considering them with the other *An. coluzzii* populations across West Africa from the Ag1000G, the PCA showed that the top three PC axes captured a disproportionate amount (~9%) of the total variance compared to the remaining PC axes (Figure S4B). The individual PC scores (Figure 1C) revealed that the Gabonese populations were well distinct from those of Angola and from those of North‐Western Africa (Burkina‐Faso, Ghana, Ivory Coast, Guinea) in the plan of the two first PC axes. The third axis further split the urban LBV mosquitoes from those of LLP. In contrast, the two proximate LLP populations could not be discriminated (Figure 1C), at least based on the set of freely recombining and unlinked SNPs used to study the genetic structure. The genetic ancestry analysis of ADMIXTURE conducted on Gabonese *An. coluzzii* populations together with those of the *Ag1000G* project revealed a similar genetic structure as the PCA. Three main genetic clusters (*K* = 3) were identified as best‐fitting solution, which minimized the cross‐validation error rates (Figures 1D and S5). These three genetic clusters were composed of *An*. *coluzzii* mosquitoes from Gabon, Angola, and NW Africa. However, additional sub‐structure was clearly visible at higher K values and consistent with the PCA and previous results from the (Ag1000G Consortium 2020). In fact, up to six distinct genetic groups were identified including Angola, LBV, the two LLP populations identified as a single genetic cluster, Burkina‐Faso, Ghana, and Ivory Coast and Guinea; the latter two also shared very similar genetic ancestries.

Various evolutionary history may explain this genetic structure, as underlined by Lawson et al. (2018). Thus, we explored further the genetic ancestry relationships among the different populations of *An. coluzzii*, their most likely branching patterns, and determined whether some may descend (or not) from admixture event(s) using *AdmixtureBayses* (Nielsen et al. 2023). The best‐fitting population graph (Figure 1E) collected 95% of the posterior probability and exhibited no admixture event among populations of *An. coluzzii*. All the nodes on the graph were highly supported with posterior probabilities > 98%. The graph suggested that the populations from NW African localities (Burkina‐Faso, Ivory Coast and Ghana) were all closely related to each other, as shown by the low estimates of genetic drift values along the branches of the graph and as suggested in the PCA and previous studies (Ag1000G Consortium 2020). Populations from Central Africa (Angola and Gabon) were also more closely to each other than those from NW Africa, as shown by the strong support on the node *n4*. Interestingly, *AdmixtureBayses* analysis suggested that populations from LBV and Angola were more closely related to each other than with the two LLP populations (node *n1*). Nevertheless, both Angola and LBV displayed elevated genetic drift values from each other, suggesting important differentiation between them as noted in the PCA and *Admixture* analyses (Figure 3C,D). The two LLP localities were very closely related to each other with very low drift values from the ancestral node (< 0.005, *n3*). They coalesced deeper in the tree with the ancestor of the (Angola, LBV) on node *n4*.

Genetic differentiation expressed as *F*_ST_ values among population pairs recovered similar genetic structuration as captured by the PCA, ADMIXTURE, and *AdmixtureBayes* analyses. Comparisons involving the Gabonese populations displayed intermediate *F*_ST_ values (0.05) for LBV versus LLP populations and no differentiation for rural and sylvatic LLP (*F*_ST_ ≈ 0, Figure S6). Aside from Gabon, all populations from NW Africa displayed shallow genetic differentiation with significant *F*_ST_ values observed for populations from Burkina Faso. Overall, as expected, the highest *F*_ST_ values were observed when comparing mosquitoes from Angola and those from NW Africa (*F*_ST_ > 0.12).

One consistent observation identified by all analyses so far was that samples from the two proximate LLP localities (i.e., village and sylvatic) remained nearly undistinguishable at the presumably nearly neutral unlinked SNPs suggesting they belong to a same genetic pool (Figures 1C,D, and S5). We tested formally this hypothesis by comparing the observed joined site‐frequency spectrum (jSFS) with a simulated jSFS expected under panmixia (obtained by permuting sample labels, see Materials and Methods) using *δaδi* (Gutenkunst et al. 2009). The observed jSFS did not statistically depart from the null distribution generated by 1000 permuted jSFS (*p*‐value > 0.45; Figure S7), confirming the genetic homogeneity observed with the PCA, ADMIXTURE, *F*_*ST*_, and the population graph of *AdmixtureBayes*. A similar analysis performed between populations from Libreville and either of the village and sylvatic LLP areas shows highly significant differences (*p*‐value < 10^−26^ for each test; Figure S7). Accordingly, average *F*_ST_ values between populations from LBV and either the rural or sylvatic areas of LLP were 0.05, one order of magnitude higher than between the two localities in La Lopé (*F*_ST_ = 1.7e‐4, Figure S6).

### 3.3 Reduced Diversity and Increased Autozygosity in Gabonese Mosquito Populations Typical of *An. coluzzii* in Central Africa

**FIGURE 2:**
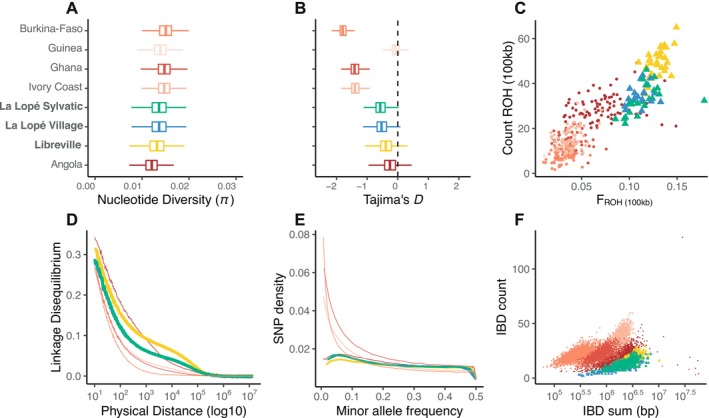
Genetic diversity of the African populations of *An. coluzzii*. Boxplots showing (A) the nucleotide diversity (π) and (B) the Tajima's *D* estimated in 10 kb non‐overlapping windows. (C) Count and frequency of the runs of homozygosity (ROH) ≥ 100 kb observed in the individual mosquitoes. Each point represents an individual mosquito. (D) Decay in linkage disequilibrium (*r* ^*2*^) as a function of the physical distance between SNPs. (E) Minor allele frequency spectrum (MAF). (F) Scatterplot of the count versus the sum of runs of identity by descent (IBD) between individuals, with each dot representing a pair of individuals drawn from the same population.

We characterized further the genetic diversity of the populations in the three Gabonese localities in comparison with those from the Ag1000G using a variety of summary statistics, including the nucleotide diversity (*π*), Tajima's *D*, long runs of homozygosity (ROH), LD‐decay, minor allele frequency, and length and count of identity‐by‐descent (IBD) segments in the genome (Figure 2). Overall, these summary statistics showed that the three Gabonese populations displayed very similar patterns to the Angolan one in Central Africa. In contrast with *An. coluzzii* populations from NW Africa, populations from Gabon and Angola displayed lower nucleotide diversity, Tajima's *D* values closer to 0, slower LD‐decay, low amounts of rare frequency variants in their SFS's, larger proportions of individual genomes covered by long runs of homozygosity (*F*_ROH_ > 100 kb), and longer inter‐individual IBD tract lengths (Figure 2). All these distinctive patterns of genetic diversity suggest that the population dynamics and history of *An. coluzzii* are very distinct in Central Africa compared to those in NW Africa, with populations of smaller effective population size (*Ne*), with possibly more stable demography with smaller *Ne* values or strongly fluctuating *Ne* size, also inducing some level of inbreeding (*F*_ROH (100kb)_ ≥ 0.10), even if this remains very small compared to strongly bottlenecked populations previously reported in *An. gambiae* such as in Mayotte Islands (*F*_ROH (100kb)_ ≥ 0.2; see figure 4 in The Ag1000G Consortium 2020). Of interest is the LBV population which displayed a slightly higher inbreeding level compared to the sylvatic and rural populations of LLP as suggested by the higher *F*_ROH_ values (*F*_ROH‐LBV_ ≈ 0.15 versus *F*_ROH‐LLPV_ ≈ 0.10), slightly higher Tajima's *D* values, slower LD‐decay, and smaller nucleotide diversity. This is consistent with smaller and possibly more fluctuating population *Ne* value in LBV than the mosquitoes from LLP.

### 3.4 Small and Relatively Stable Demographic History of Gabonese Populations

**FIGURE 3:**
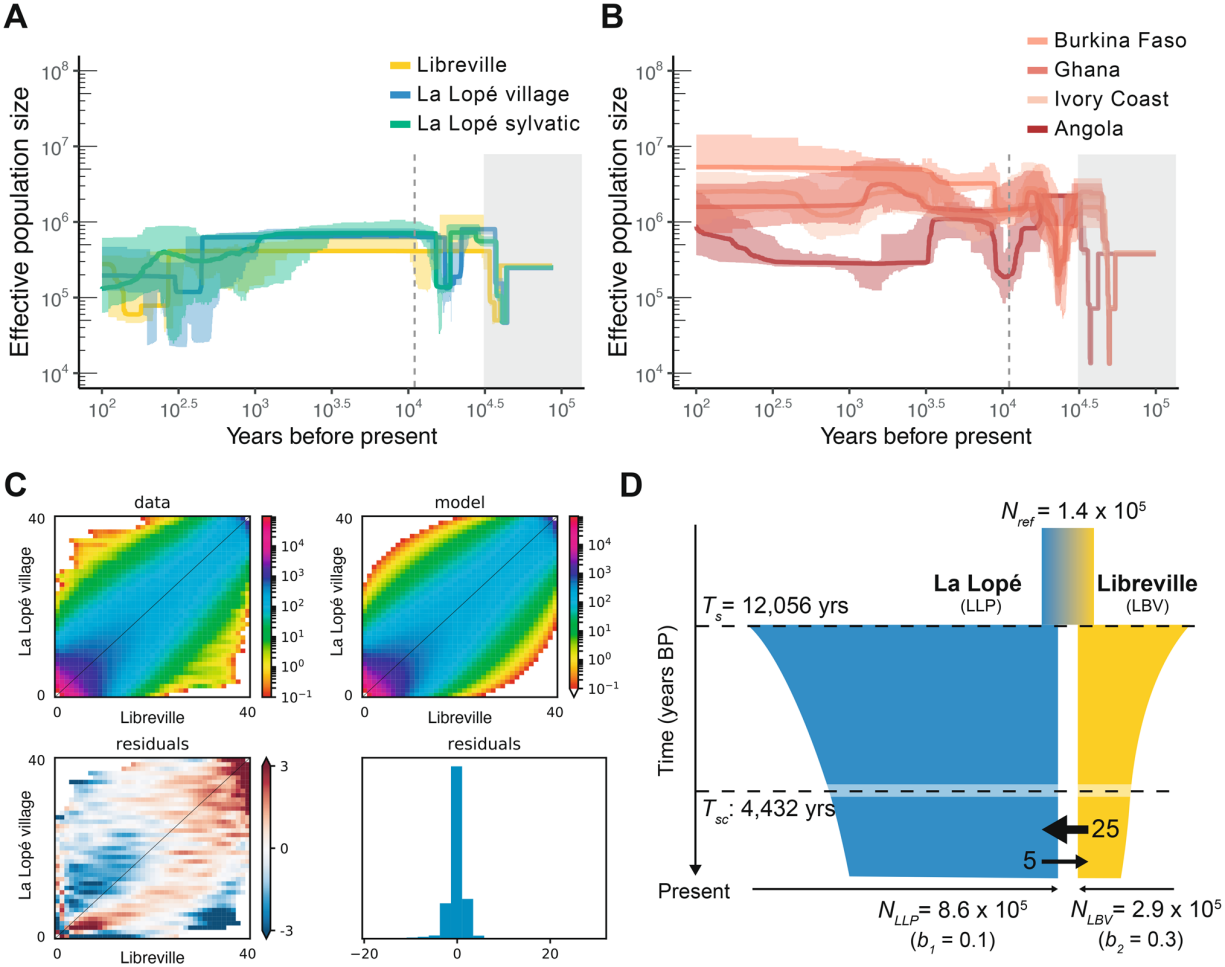
Demographic histories of the African populations of *An. coluzzii* estimated from genetic data. (A, B) Historical changes in effective population size (*Ne*) were inferred using *Stairway Plots* 2 (Liu and Fu 2020) for the three Gabonese populations (A) and for populations of the Ag1000G consortium (2017) from West Africa and Angola provided here as a comparison (B). The *Ne* values for each population were rescaled using a generation time (g) of 11 years and a mutation rate (*μ*) of 3.5 × 10^−8^ per site and per year. The main‐coloured lines show the median estimates and light shaded areas represent 95% confidence intervals. In both panels (A) and (B), the grey dashed line indicates the split time between populations from La Lopé and Libreville estimated from the *∂a∂i* analysis (see panel D and Table 1), and the grey rectangle indicates the range of split time estimates between *An. coluzzii* and its closest relatives, *An. gambiae*, from the literature (The Ag1000G Consortium 2017; Thawornwattana et al. 2018; Müller et al. 2021). (C) Results of *∂a∂i* (Gutenkunst et al. 2009) 2‐populations analysis. The top panel represents the observed joint site frequency spectrum (jSFS) for Libreville versus La Lopé village along with the one expected under a secondary contact model (SC + G) fit (top right panel) and distributions of the residuals (bottom panel). (D) Schematic representation of the demographic model best fitting the jSFS in the *∂a∂i* analysis. This SC + G model involves an allopatric split at time *T*_*s*_ between the populations from La Lopé and Libreville, followed by a secondary contact at time *T*_*sc*_ restoring asymmetric geneflow, and exponential population size changes with *b*_1_ and *b*_2_ population rates for La Lopé and Libreville, respectively (see Table 1 for details on parameters estimations).

We estimated the demographic history of each population of *An. coluzzii* in Gabon, comparatively with those from the *Ag1000G*, using *Stairway plot* v2 (Liu and Fu 2020) based on the unfolded (*a.k.a*. polarized as ancestral versus derived allelic state) SFS of the putatively neutral and recombining portion of chromosome 3 (Figure 3A,B). The two populations from LLP shared similar historical trajectories in *Ne* variation, with 2 mild and old bottlenecks, one *ca*. 400 k generations ago (or 40 k years ago kya), and another one *ca*. 200 k generations ago (or 20 kya). *Ne* values remained relatively stable since the past 10 k years, with however evidence of a slight decline during the past 1000 years. Noteworthy was the population from LBV, which displayed consistently lower effective sizes compared to the rural and sylvatic LLP populations, in line with the genetic diversity estimates (Figure 2). Even if confidence interval was large at very recent time scale, Ne values in the LBV population marked a one order magnitude reduction during the past 300 years compared to LLP populations. The effective population sizes of the three Gabonese populations (Figure 3A) were also comparable to those observed in Angola, and were at least one order of magnitude smaller than those from NW Africa (Figure 3B), also consistent with genetic diversity estimates and previous studies (Ag1000G Consortium 2020). Interestingly, the 2 old consecutive bottlenecks detected in the Gabonese populations were also observed in Angola, and to a lesser extent, also in the populations from NW Africa (Figure 3A,B). This suggests that these ancient Ne variations likely pertain to various evolutionary processes impacting genetic diversity in the ancestral population(s) prior the split of the Gabonese populations, and almost at the same time as the split between *An. gambiae* and *An. coluzzii* (~50 kya; The Ag1000G Consortium et al. 2017; Thawornwattana et al. 2018; Müller et al. 2021). These old Ne variations may reflect changes in ancestral population structure, connectivity, and demographic (see e.g., Mazet et al. 2016).

### 3.5 Secondary Contact and Asymmetric Gene Flow Have Homogenized Gabonese Populations

To better understand the isolation history between the populations from LBV and LLP, we tested which isolation models best fitted the observed unfolded joined‐SFS (jSFS) between the two populations using *δaδi* (Gutenkunst et al. 2009) and the framework of Rougeux et al. (2017). We compared 8 distinct models of population isolation differing in terms of *Ne* variation (constant vs exponential changes, ‐G), and in terms of gene flow history contrasting strict isolation without gene‐flow (‐SI), isolation with continuous migration (‐IM), ancestral migration (‐AM), or secondary contact (‐SC). The best fitting model displaying the lowest log‐likelihood scores and best AIC criterion identified the scenario implying a secondary contact with population‐size change (SC + G; Figure S8 and Table S2). This demographic scenario suggests that the isolation between LBV and LLP involved a period of allopatric divergence followed by a secondary contact (SC), with a slight asymmetric gene flow predominantly from LBV into LLP, and a mild exponential decline in effective sizes for both populations, yet slightly stronger in Libreville city (LBV) than in La Lopé (LLP) (Figure 3C). The most likely parameter estimates for this SC + G model (Figure 3D, Tables 1 and S2) suggest that the populations from Libreville city (LBV) and La Lopé (LLP) split *ca*. 12 k years ago (*T_s_*) from an ancestral population (*N_ref_*) into two populations, each one with distinct *Ne* values, respectively of 292,460 and 865,159 individuals for LBV and LLP. The two populations would have remained isolated until *ca*. 4,432 years ago (*T*_*sc*_), time at which a secondary contact would have restored an asymmetric gene flow with five times more effective migrants from LBV into LLP than in the reverse direction (Figure 3D, Tables 1 and S2).

**TABLE 1:** Best fitted demographic model of population split with secondary contact between LPP and LBV populations, involving also exponential population size changes within each population (SC + G model), as estimated from DADI.

SC + G	Optimized parameters in biological units	Lower 95% CI	Upper 95% CI
*N*_*ref*_	142,332.55	128,617.05	156,048.05
*N*_*1‐LLP*_	865,159.02	611,580.53	1,118,737.51
*N*_*2‐LBV*_	292,460.51	222,094.35	362,826.68
*T*_*s*_	12,056.12	6032.30	18,079.94
*T*_*sc*_	4431.69	639.16	8224.22
*m*_*12*_	0.003%	0.002%	0.004%
*m*_*21*_	0.002%	0.001%	0.002%
*nb_m*_*12*_	25.10	12.16	42.67
*nb_m*_*21*_	4.80	2.49	7.86
*b*_*1*_	0.10	0.08	0.12
*b*_*2*_	0.31	0.19	0.42

*Note:* The most likely parameter values and their 95% confidence intervals are provided in biological units (see Table S2 for original estimates). Effective size values are in number of individuals, split time (*T*_*s*_) and time of secondary contact (*T*_*sc*_) between the two populations are in years before present, migration rate (*m*_*ij*_) is the fraction of the population *i* coming from *j* per generation, and the effective number of migrants per generation (*nb_m*_*ij*_) is the number of individuals from population *i* coming from *j* per generation. The rate of population size change within each population is characterized by an exponential change with a lambda parameter *b* (See also Figure 3D and Table S2).

### 3.6 Genome Scans Suggest Contrasted Regimes of Selection Across Urban, Rural, and Forested Environments in Gabon

**FIGURE 4:**
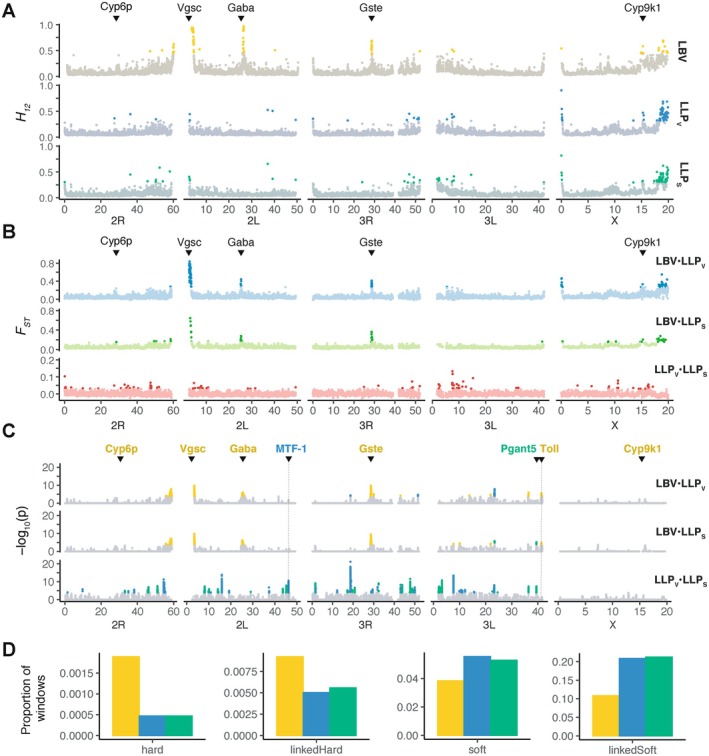
Signals of positive selection among Gabonese populations. Genome‐wide scans for (A) *H_12_*, (B) *F*_ST_ and (C) XP‐EHH *p*‐values statistics. For *H_12_* and *F*_ST_, the top 1% windows are highlighted in bright colours. In XP‐EHH plot, each dot is coloured according to its significance, with grey dots indicating non‐significant SNPs and coloured dots representing significant value (*p*‐value < 1e^−4^) suggesting positive selection. Key candidate genes discussed in the text are highlighted. Note that the y‐axis scales differ between rows. (D) Proportion of the overall genomic windows (*N* = 12,693) classified within the four different class sweeps in the *diploS/HIC* analysis. Colour code of the populations is consistent with Figure 1.

We screened the genome for evidence of positive selection at genes or genomic regions that may have contributed to local adaptation across the sylvatic, rural, and urban environments. We first conducted genome scan within and between populations based on summary statistics relying on haplotype homozygosity (*H*_12_; Garud et al. 2015), differences in allele frequency (*F*_ST_—based statistics), and differences in long‐range haplotype homozygosity (XP‐EHH statistic, Sabeti et al. 2007). Comparisons between the urban LBV and either rural or sylvatic LLP populations (LLP_V_ or LLP_S_, respectively) revealed a few clear and strong signals of positive selection in the urban population at *H*_*12*_ (Figure 4A), *F*_ST_ (Figure 4B), and XP‐EHH (Figure 4C) statistics. Those selection footprints were found centered mostly around well‐known genomic regions harbouring insecticide resistance genes, jointly detected by *H*_*12*_, *F*_ST_, and/or XP‐EHH statistics. The three statistics pointed to strong selection signals at the *Voltage‐Gated Sodium Channel – (VGSC)* in the pericentromeric region of chromosome 2L, the resistance to dieldrin (*Rdl*) locus that encodes the GABA receptor (*GABA*) in chromosome 2 L, and the Glutathione S‐transferase epsilon clusters (*GSTE* genes) on chromosome 3R. *H*_*12*_ and *F*_ST_ statistics also identified the cytochrome P450 *Cyp9k1* on chromosome X. Additionally, away from the well‐known insecticide resistance genes, a few other genomic regions exhibited also significant evidence positive selection in the urban LBV population. Among them, one stretch between positions 41,275,000 and 41,500,000 on the chromosome 3L consistently stood out through the comparisons between LBV with both LLP_V_ and LLP_S_ population in the XPEHH scan (Figure 4C). The four annotated genes in this region included the gene *AGAP012385* encoding for a Toll‐like receptor signalling pathway known to mediate anti‐pathogen defence, including against *Plasmodium* (Clayton et al. 2013). Twenty‐six significant SNPs were found in the 5 kb upstream and downstream regions of that gene.

Unlike the strong and very localized signature of positive selection observed between Libreville and La Lopé, selective footprints detected between the village and sylvatic area of LLP (LLP_V_ vs. LLP_S_) were identified mostly by XP‐EHH statistic, which displayed clear selection signals, but more disperse across the autosomes. Only a small subset of the XP‐EHH signals were also detected by *H*_*12*_ and *F*_ST_ scans (Figure 4A–C). This suggests that the selection signals likely involve primarily soft selective sweeps acting on standing genetic variation (SVG), and which thus display more limited differences in allele frequency with little impact on *F*_ST_ values. In total, 44 significant regions were detected using the XPEHH statistics and exhibited strong positive signatures of selection and scattered across the autosomes between the village and sylvatic area of LLP. These 44 outlier regions included a total of 118 candidate genes (Table S3). Although gene ontology (GO) enrichment analysis did not reveal any significant enrichment in specific gene functions, some candidate genes stood out from the group. The metal response element‐binding Transcription Factor‐1 (*MTF‐1*, AGAP007377) was found to show clear signals of selection in LLP village, but not in the sylvatic LLP populations. This gene encodes for a zinc finger protein involved in the detoxification of non‐essential, toxic heavy metals, such as cadmium, mercury, and silver. In parallel, in the sylvatic LLP population, the gene *pgant5* (AGAP012256) responsible for post‐translational modification was found under strong positive selection in both comparisons with the village and the city. In *Drosophila*, this gene is a member of a large gene family known to participate in many aspects of development and organogenesis, notably in the upkeeping of a proper digestive system acidification (Tran et al. 2012).

Annotating the SNP effect using *snpeff* (Cingolani 2022), SNPs identified as outliers with XP‐EHH scans shows that variant sets were significantly different in the nature of their annotation for the three pairwise population comparisons across the three types of environment (Figure S9A, 𝜒^*2*^ test = 538.562, *p‐value* < 1e^−5^). Selected variants distinguishing the urban LBV from the rural LLP area (LLP_V_) were found enriched in synonymous SNPs, while variants distinguishing the village from the sylvatic LLP populations (LLP_V_ vs. LLP_S_) were found enriched at intergenic regions (Figure S9B). Together, our results based on genome scans with the three summary statistics (*H_12_*, *F*_ST_, and XP‐EHH) highlight the contrasted nature of the positive selection signal across our three pairwise comparisons. On one hand, between the urban LBV population and either the rural or sylvatic LLP populations, strong selection signals are detected, extremely localized at very few loci. On the other hand, between the village and the sylvatic LLP populations, the signal is more spread out through the genome.

### 3.7 Model‐Based Genome Scans Based on Supervised Deep Learning Confirm the Contrasted Selection Regimes Across Environments in Gabon

We explored further the hypothesis that urban population from LBV and the two proximate localities in the LLP National Park exhibit distinct selective regimes: the first one being dominated by hard‐selective sweeps, and the two others by soft‐sweeps on the SGV. For that purpose, we used the model‐ and simulation‐based supervised deep learning technique of *diploS/HIC* (Kern and Schrider 2018). High level of convergence was observed for the summary statistics of our simulated dataset compared to the empirical data under the different selection categories (Figure S10). For each of the 12 descriptive statistics composing the feature vectors used by *diploS/HIC* to classify genomic windows, a good convergence was observed between simulated and empirical data, with the exception of a slight reduction in nucleotide diversity (π) in the simulated datasets compared to the observed data, regardless of the population of interest. This is likely due to the fact that our simulations framework is conducted within population, ignoring thus gene flow from other populations. Nevertheless, beside the genetic diversity slightly under‐estimated by our simulation, all the other statistics that are the most important contributors in detecting selective signals (see Kern and Schrider 2018) fitted very well between simulations and observed data.

The Convolutional Neural Network (CNN) classifier performed well in distinguishing the five selection categories based on our simulations (Figure S11). Hard sweeps were correctly identified with 76% accuracy and minimal confusion with neutral windows (< 3%). Linked‐hard sweeps also showed high classification accuracy (71%), while soft sweeps were detected with 61% accuracy. Importantly, over 80% of soft sweeps were recognized as under positive selection, suggesting that there is little risk of missing a selection signal from soft sweeps. Neutral windows were accurately classified in 77% of the time, limiting false positives in our study. Linked‐soft sweeps proved most challenging, with only 47% accuracy, as expected given their limited genomic signatures, with most of the confusion occurring with neutral windows (~26% misclassification; Figure S11). Overall, *diploS/HIC* demonstrated high sensitivity and precision in detecting and differentiating windows under positive selection in our study design.

The results of diploS/HIC (Figure 4D) confirmed the contrasted selection regimes across the three types of environments suggested by the genome scans for the three descriptive statistics (*H_12_*, *F_ST_* and XP‐EHH) (Figure 4A–C). Indeed, *diploS/HIC* showed that most of the genomic windows identified as evolving under positive selection in the urban LBV population were mostly classified as hard or linked‐hard selective sweeps. In contrast, genomic windows evolving under positive selection in the rural or sylvatic populations of La Lopé (LLP_V_ and LLP_S_) were primarily identified as soft or linked‐soft selective sweeps (Figure 4D). Together, both genome‐scans approaches based either on descriptive statistics or model based machine learning support the different modes of local adaptation acting on the urban LBV population compared to those from the LLP National Park: a few *de novo* mutations were selected in the LBV city due to the strong selection pressure applied by insecticide or polluted environments, while positive selection on SGV is the most frequent mode of adaptation across less anthropized populations in the La Lopé National Park.

We obtained limited overlap in candidate genes or genomic windows identified as under positive selection between the selection scans based on the summary statistics (*H_12_*, XP‐EHH, and *F*_ST_) and *diploS/HIC* (Table S3) because the two type of methods do not operate at the same resolution. *DiploS/HIC* required large genomic windows of 110 kb to apply the CNN classifier, while the descriptive statistics can be conducted in smaller windows (1 kb, 10 kb or 20 kb) or even at the SNP level. Nevertheless, we found that most of the selective sweeps detected by haplotype‐based, frequency‐based, and *diploS/HIC* analyses in the urban LBV population were also identified as hard‐ (or linked‐hard) selective sweeps and involved the known insecticide resistance and immune‐related genes (Table S3). In contrast, evidence of positive selection between in the village and sylvatic LLP populations were primarily soft‐ (or linked‐soft) selective sweeps scattered along the autosomes (Figure 4A–C).

## 4 Discussion

Understanding the evolutionary mechanisms by which populations and species adapt locally along heterogeneous habitats is a fundamental question in ecology and evolution. Here, we evaluated how and by which population genetic processes a highly anthropophilic malaria‐vector mosquito species, such as *An. coluzzii*, most likely colonized and adapted to varying levels of human presence in Gabon, from the densely populated urban area of Libreville to rural and forested areas deprived of any permanent human settlement in the La Lope National Park. To this end, we used a combination of population genetic approaches to analyse deep whole‐genome sequencing data from 77 individual mosquitoes. We investigated the genetic structure and demographic history of these Gabonese populations of *An. coluzzii* in comparison with other African populations (The Ag1000G Consortium 2020), as well as the selective forces driving local adaptation to the different habitats.

### 4.1 Populations of *An. coluzzii* From Gabon Exhibit Singular Genetic Signatures

Consistent with previous studies (The Ag1000G Consortium 2020; Campos et al. 2021; Pinto et al. 2013), we identified three major genetic groups across *An. coluzzii'*s distribution range: (i) the North‐West African group composed of the mosquitoes from Burkina‐Faso, Guinea, Ivory Coast, and Ghana; (ii) the Central African group composed of the Gabonese populations; and (iii) the South‐Western African group with Angola (Figure 1C). These clusters coincide with the transitions between the central African rainforest belt and the Western northern and southern savannah biomes (Pinto et al. 2013; Tene Fossog et al. 2015). The Congo rainforest block has been suggested to constitute a potent barrier to gene flow (Pinto et al. 2013; Tene Fossog et al. 2015). Previous works from Campos et al. (2021) showed that the Gabonese *An. coluzzii* specimens clustered with those from the neighbouring country, Cameroon, supporting a Central African cluster of *An. coluzzii* distinct from the others. Our analyses underlined the distinctiveness of the Gabonese LLP mosquitoes from more southern populations in Angola. Nevertheless, population‐graph analysis suggested that coastal populations (i.e., Libreville and Angola, Figure 1E) shared together a more recent common ancestor than with the North‐West African group, north of the Congo‐basin. This result is in agreement with previous works that evidence a subdivision between coastal and inland populations of *An. coluzzii* (Slotman et al. 2007; Tene Fossog et al. 2013). Moreover, the genetic characterization of the three populations from Gabon revealed higher levels of homozygosity compared to North‐Western populations, consistent with smaller and more isolated populations (Figure 2). This result confirms that even at the “local” scale, the transition from coastal to rainforest area is associated with strong changes in the genetic make‐up of *An. coluzzii* mosquitoes, as we can observe between Libreville and La Lope that are only ~250 km away from each other (Figure 1D).

### 4.2 Historical Rainforest Evolution Likely Drove Human and Mosquitoes' Expansion in Central Africa

Our demo‐genetic analyses suggest that the Gabonese populations from Libreville (LBV) and La Lopé National Park (LLP) split *ca*. 12,000 years ago and later came back into contact *ca*. 4,400 years ago (Figure 3D). The population split time coinciding with the peak of the African humid period and rainforest expansion toward the tropics (Malhi et al. 2013; Willis et al. 2013). These climate changes likely impacted human movements between Central African and other human African populations (Batini et al. 2011; Laval et al. 2019; Lopez et al. 2018, 2019; Patin et al. 2009; Verdu et al. 2009), as well as the population dynamic of major malaria mosquitoes due to their close relationship with humans.

Interestingly, the secondary contact around 4,400 years ago that restored gene flow between the coastal urban LBV population and the inland LLP mosquito populations aligns with the human history of large‐scale migrations of Bantu‐speaking peoples from the coast through the Central Africa rainforest (Batini et al. 2011; Laval et al. 2019; Lopez et al. 2018, 2019; Patin et al. 2009; Verdu et al. 2009). Fueled by agriculture development, Bantu speaking human populations migrated through the Central African rainforest around 4400 year ago, spreading the Bantu culture toward Central and South Africa (Koile et al. 2022). It is thus very likely that large‐scale human movements facilitated secondary contact between isolated mosquito populations, as estimated in our *δaδi* analyses (Figure 3D). The post‐glacial expansion (20 k years ago) of the Bantu speaking farmers, and their colonization toward Central and South Africa (4000 to 5000 year ago) likely reconnected isolated mosquito populations from the coastal and inland areas. In agreement with this scenario, the La Lopé National Park has a rich history of ancient human trades and migration corridor along the Congo Basin for Pygmy tribes, Bantu, and other people (UNESCO World Heritage Centre 2016). These extensive historical human movements likely facilitated the dispersion and interconnectedness of human‐specialized mosquitoes. Also consistent with this scenario is the timing of emergence within the genome of the forest‐dwelling pigmy tribes of Central Africa of the genetic mutation causing sickle‐cell anaemia (a.k.a. drepanocytosis) which provides a strong selective advantage against malaria. This mutation emerged at least 20 k year ago in Africa primarily in the genome of the Bantu's farmers, and was only introduced ~4 k to 5 k years ago into the rainforest pygmy hunter‐gatherers of Central Africa, after admixture event between the two (Laval et al. 2019).

It is important to emphasize that our interpretation of the historical events shaping the evolution of Gabonese *An. coluzzii* populations remains correlative and speculative, as is typical in phylogeographic studies. Our *δaδi* results (Figure 3D) are based on simplified population genetic models and on point estimates—such as mutation rates and generation times—to convert model parameters into biological units. Although these estimates are widely accepted for *An. gambiae* and *An. coluzzii* and have been broadly used in the literature, they are still uncertain and may vary geographically. While the correspondence between our estimates and historical events provides an explanatory framework for our demographic inferences, these remain hypothetical and should be interpreted with caution. Future research with larger sample sizes, improved estimates of generation time and mutation rates, as well as more realistic demographic models, may help refine our understanding of the evolutionary history of *An. coluzzii*.

### 4.3 Interplay Between Urban Adaptation and Insecticide Resistance in *Anopheles coluzzii*

In contrast to the previously reported “shallow to moderate” population substructure among *An. coluzzii* populations within the North‐West African group (*F*_ST_ ≤ 0.007, Figure S6; The Ag1000G Consortium 2017, 2020), we observed moderate to high genetic structure within the Central African cluster at short geographic distance (*F*_ST_ = 0.047; Figure S6). The urban coastal mosquito population from LBV was genetically highly distinct from LLP populations located only 250 km inland. These urban mosquitoes displayed a population dynamic and demography that contrasted strongly with the population from the more rural and sylvatic environment of the La Lopé National Park (LLP) (Figure 2). The urban LBV population was more inbred, it displayed stronger linkage disequilibrium, higher autozygosity, and a three‐fold reduced effective population size compared to those from LLP (Figure 2). Such genetic contrasts may reflect differences in human activities, such as vector control measures and/or pollution level in breeding sites between rural and urban areas (Longo‐Pendy et al. 2021; Vargas‐Chavez et al. 2022). Consistent with this hypothesis, we found few but strong selection signals identified in the genome of the LBV populations, mostly composed of hard (and linked‐hard) selective sweeps at sites well‐known for insecticide resistance and detoxification processes (Figure 4, Table S3). Among them, we identified *kdr* mutations in *VGSC* of mosquitoes conferring resistance to pyrethroid insecticides (Martinez‐Torres et al. 1998; Ranson et al. 2000; Davies et al. 2007; Dong et al. 2014; Clarkson et al. 2021; Nagi et al. 2025); the *Rdl*—GABA‐gated chloride channel subunit gene, a locus with prior evidence of positive selection and/or an association with dieldrin insecticide resistance in *Anopheles* mosquitoes (Du et al. 2005; Grau‐Bové et al. 2020; Nagi et al. 2025); and the GSTE—Glutathione S‐transferase epsilon gene cluster (Mitchell et al. 2014; Nagi et al. 2025). The LBV populations also harboured strong selective signal at the toll‐like receptor signalling pathway known to mediate anti‐pathogen defence, including against *Plasmodium* (Clayton et al. 2013). The African cities have been spared of *Anopheles* until recently (Robert et al. 2003). During the last two decades, *An*. *coluzzii* has overcome the constraints linked to polluted breeding sites and it is now the predominant *Anopheles* species in the cities of Central Africa (Doumbe‐Belisse et al. 2021; Kamdem et al. 2017; Longo‐Pendy et al. 2021; Tene Fossog et al. 2015). As we observe in our study, this adaptation is likely associated to detoxification and insecticide resistant genes as previously documented (Antonio‐Nkondjio et al. 2015; Kamdem et al. 2017). Similar type of adaptation to urban environment were also reported in other mosquitoes such as *An. stephensi* (Tiwari et al. 2010) and *Aedes aegypti* (Kwame Amlalo et al. 2022). Therefore, urbanization tends to favour mosquito populations that are resistant to pollution and insecticides, which become less affected by actual vector control measures. Interestingly, the fact that a toll‐like immune receptor is also strongly selected in the urban population raises the question about how urban adaptation and pollution can affect vector competence to *Plasmodium*, a key aspect to understand and prevent urban malaria (Doumbe‐Belisse et al. 2021).

Given the asymmetric gene flow from LBV into the inland LLP populations, we might have expected to find insecticide resistance alleles in the genomic background of the LLP population. However, no such selection signal was observed. Previous studies reported that insecticide resistance alleles carry fitness costs, including higher metabolism, reduced body size, and shorter lifespan which can lead to their loss in the absence of insecticide pressure (Amaya Romero et al. 2024; Ingham et al. 2017, 2021; Lucas et al. 2023; Oliver and Brooke 2016). For instance, such fitness effects led insecticide alleles to disappear in mosquitoes colonies (Ingham et al. 2021). In La Lope village, no vector control strategies are implemented. Our results suggest that resistant alleles were not detected in LLP populations, either because they are absent due to fitness costs or because they remain at very low frequency undetected by our limited sample size of ~20 individuals per site. Nevertheless, gene flow from neighbouring populations such as LBV could rapidly introduce resistance alleles under insecticide selection pressure.

### 4.4 Standing Genetic Variation Is Key to Local Adaptation

In contrast to the coastal urban population of LBV, *An. coluzzii* from La Lopé (LLP) village and sylvatic areas displayed higher genetic diversity, slightly lower Tajima's *D*, lower autozygosity and LD in their genomes. These genetic features suggest that the LLP populations have remained more stable in size for a longer time, and without any major bottleneck in a recent past like those suspected in the coastal LBV population in response to adaptation to polluted urban environment (Figure 2). This was supported by the *Stairway Plot* and *δaδi* analyses which reported only minor variations in effective population in the LLP population since its split from the LBV population (12 kyr or 120 thousand generations ago) (Figure 3). Very ancient bottlenecks were detected by *Stairway Plot* between 30 k and 50 k years ago (thus more than 300 k to 500 k generations ago) in the Gabonese populations, but also more widely in *An. coluzzii* across Africa (Figure 3A,B). These bottlenecks occurred far before the split between LBV and LLP, and the oldest ones coincided with the split time estimated between *An. coluzzii* and its closest relative, *An. gambiae* (Ag1000G Consortium 2017, 2020; Thawornwattana et al. 2018; Müller et al. 2021; Figure 3A,B). These ancient demographic changes thus reflect evolutionary processes that took place in the ancestral populations, such as ancestral population structuration, demographic fluctuations, or selection (see for ex. Mazet et al. 2016).

Importantly, the patterns of genetic diversity and stable effective sizes in the populations from La Lopé village and sylvatic neighbourhoods may also suggest that they are connected to other forested populations, across villages and unsampled sylvatic places. Despite the differences in hosts or breeding sites between both habitats at La Lope, the mosquitoes from the village and the sylvatic area were part of a same genetic pool (Figures 1C and S4). All analyses conducted to assess population genetic structure and differences in genetic diversity failed to discriminate mosquitoes from these two locations when using putatively neutral and unlinked SNP genetic markers (Figures S6 and S7). At a short geographic scale (~10–15 km), panmixia is expected given the known dispersal capacity of *An. coluzzii* (Ayala et al. 2013; Costantini et al. 1999). Although strong isolation by distance (IBD) has been reported in this species across broader regions of Africa, particularly in the NW savannah and across major biogeographic barriers such as the savannah–rainforest transition (Ag1000G Consortium 2020; Lehmann et al. 2003; Pinto et al. 2013), such patterns emerge at larger spatial scales. Indeed, Battey et al. (2020) showed that the geographic origin of individual mosquitoes could be predicted from genetic data with an average precision of 5 km (median 36 km), reflecting broad‐scale IBD. However, at fine scales such as 10 km, gene flow is likely sufficient to maintain panmixia, unless other evolutionary processes (e.g., selection) intervene.

The selection regime between village and sylvatic populations at La Lopé was dominated by soft (and linked‐soft) selective sweeps on the SGV (Figure 4), in contrast to a selective regime dominated by few hard‐selective sweeps in the urban LBV population. The mode of adaptation of the LLP populations mostly involved selection on the SGV (and possibly also rapidly recurring beneficial mutations appearing on different haplotypes; Charlesworth and Jensen 2021; Garud 2023; Johri et al. 2022). Adaptation from SVG has been shown to dominate also in other species like *Drosophila*, human and other organisms, even if some debates persist (Schrider and Kern 2017; Harris et al. 2018; Stephan 2019; Charlesworth and Jensen 2021; Feder et al. 2021; Garud et al. 2021; Johri et al. 2022). This predominance of soft (or link‐soft) sweeps observed between sylvatic and village LLP populations was also shown to be relatively common across the genome of *An. coluzzii* (Xue et al. 2021). By analysing the data from the Ag1000G Consortium (2017), Xue et al. (2021) reported that soft and partial selective sweeps were common place in the genome of 8 populations of *An. gambiae* and *An. coluzzii* across Africa. Nevertheless, mosquitoes from both LLP localities exhibited striking differences in selective signals marked by distinctive contrast at the cross‐population extended haplotype homozygosity (XP‐EHH) scattered across their genomes, revealing selection pressures despite gene flow (Lenormand 2002).

When comparing populations from the village and sylvatic areas of La Lopé, most of the candidate genes or genomic regions under positive selection were identified using the cross‐populations extended haplotype homozygosity (XP‐EHH statistics), with little overlap with the signals detected by the *H*_*12*_ and F_ST_ statistics (Figure 4A–C). This discrepancy suggests that the selection signals are most likely due to soft or partial selective sweeps, which involve only subtle changes in allele frequencies. The XP‐EHH test can detect selective sweeps in which the selected allele has risen to high frequency or fixation in one population, but remains polymorphic in the population as a whole (Sabeti et al. 2007). Under this circumstance, XP‐EHH can detect signals in regions that do not display strong outlying *F*_ST_ values. *F*_ST_‐based selection scan is meant to detect signals of excessive levels of genetic differentiation, which would typically capture hard selective sweeps, such as those detected at known insecticide resistance genes discriminating the city from the rural area. However, in the case of soft sweep which implies primarily selection on the SGV, differences in allele frequency become much more subtle, and the power to detect significant outlying *F*_ST_ values drops as well (Sabeti et al. 2007; Voight et al. 2006). Since the populations from the village and sylvatic area of La Lopé (LLP) are derived from the same genetic pool and exhibit similar demographic histories and LD‐decay, the sensitivity of the cross‐populations extended haplotype homozygosity (XP‐EHH statistics) is maximized to identify even subtle processes of positive selection (Klassmann and Gautier 2022; Ma et al. 2015). Our model‐based *diploS/HIC* genome scans relying on supervised machine learning also confirmed that soft selective sweeps were dominating in the remote populations of La Lopé village and sylvatic areas (Figure 4D). However, as noted in the confusion matrix (Figure S7), CNN‐based approaches such as diploS/HIC generally have higher power to detect hard sweeps than soft sweeps. Soft‐sweep classifications should therefore be interpreted with more caution, particularly under complex demographic histories (Harris et al. 2018; Johri et al. 2022).

These selective signals scattered across the genomes did not reveal any major genetic enrichment signal (Table S3). As noted by Xue et al. (2021) for the populations from the Ag1000G Consortium (2017), the numerous calls of selective sweep detected may reflect complex selective dynamics at play, for example, polygenic and quantitative trait adaptation (Pritchard et al. 2010; Booker et al. 2017), balancing selection (Connallon and Clark 2013), and introgression of beneficial alleles from neighbouring unsampled populations (Ag1000G Consortium 2017, 2020). Local adaptation of the mosquitoes to an un‐anthropized habitat such as the sylvatic areas, compared to the village of the LLP National Park, likely involved many phenotypic traits that require further investigations. For instance, it would be particularly relevant to investigate whether structural variants, such as chromosomal inversions, gene copy number variations, or transposable element polymorphisms, influence mosquitoes' adaptation to non‐anthropized habitats (Battlay et al. 2018; Samano et al. 2024). These variants often induce significant adaptive shifts in ecologically important traits, making them compelling candidates for driving rapid adaptations to environmental changes. Nevertheless, such local adaptation is expected to be highly polygenic and it is unlikely that all alleles involved are newly mutated.

## 5 Conclusions

Our study has provided further evidence of the remarkable adaptability of a major malaria vector enabling it to thrive in distinct ecological settings. In Central Africa, *An. coluzzii* inhabits a wide range of environments, from highly anthropogenic and polluted urban habitats to remote rural and wild protected natural areas, such as National Parks. This study characterized for the first time, to our knowledge, a population of a major malaria mosquito capable of colonizing and thriving in areas devoid of permanent human settlement, yet in direct proximity to a village. Our findings offer new insights into how mosquitoes adapted to humans primarily through selection on their ancestral standing genetic polymorphism. Colonization and adaptation of malaria vector mosquitoes to urban areas pose a significant threat for malaria control and eradication in the coming years (Venkatesan 2024). The ability of *An. coluzzii* populations to persist in remote areas away from permanent human presence represents a considerable challenge for malaria vector control and eradication. Remote protected areas being a refuge for malaria vectors have been neglected and little is known about how it can affect vector control strategies. Further research is needed to better understand the phenotypic changes, ecological, and behavioural adaptations of these and other malaria vectors in this unique ecological scenario.

## Author Contributions

Conception: D.A., D.E.N., M.C.F; funding acquisition: D.A. and D.E.N.; biological data acquisition and management: D.A., L.B., N.R., B.M, O.A.E, M.F.N., N.M.L.P., C.P.; sequence data acquisition and management: D.E.N., J.T., L.T., M.C.F.; method development and data analysis: J.D. and M.C.F.; interpretation of the results: J.D., M.C.F., and D.A.; drafting of the manuscript: J.D., M.C.F., and D.A.; reviewing and editing of the manuscript: J.D., M.C.F., and D.A., with inputs from all the co‐authors.

## Funding

This work was supported by the French National Research Agency (anr.fr): ANR‐18‐CE35‐0002‐01—WILDING; National Institute of Allergy and Infectious Diseases, National Institutes of Health, Department of Health and Human Services, U19AI110818.

## Conflicts of Interest

The authors declare no conflicts of interest.

## Supporting information


**Figure S1:** (A) Bioinformatic workflow from the reads cleaning, mapping to a the AgamP4 PEST reference genome, and SNP genotyping and quality filtering procedures used. (B) Schematic overview of the different analysis and the input dataset used.
**Figure S2:** Sequencing coverage depth and SNP density along the genome.
**Figure S3:** Kinship analysis across the Gabonese dataset estimated with pair‐wise IBD estimator (PI_HAT) between samples in PLINK (Anderson et al. 2010).
**Figure S4:** Extended population structure analysis (A) Scree‐plot showing the variance fraction explained by each principal component of the PCA for the African *An. coluzzii* samples (combining the Gabonese and AG1000G datasets) represented in Figure 1. (B) PCA of the 77 An. coluzzii mosquitoes from Gabon retained for further analysis using biallelic unlinked SNPs from the euchromatic regions of the chromosome 3. The bar chart shows the percentage of variance explained by each principal component.
**Figure S5:** Analysis of population structure and genetic ancestry in *An. coluzzii* considering the Gabonese populations in perspective with those from the *Ag1000G*.
**Figure S6:** Pairwise population differentiations (*F*_*ST*_) among populations of *An. coluzzii*.
**Figure S7:** Test of departure from random mating expectation (panmixia) between pairs of populations performed using *δaδi*.
**Figure S8:** *δaδi* model selection based on the AIC score obtained for 8 different models and 100 replicates per model.
**Figure S9:** Population‐specific proportion and functional categories of SNPs with significant XP‐EHH values.
**Figure S10:** Goodness‐of‐fit between empirical and simulated data under the 5 different types of selection scenarios of selective sweep in the *diploS/HIC* analysis.
**Figure S11:** Graphical representation of the confusion matrix for each population obtained from the CNN classifier of *diploS/HIC*.

**Table S1:** Description of the *An. coluzzii* sampling from Gabon for which whole genome sequencing was performed and sequencing statistics.
**Table S2:** Summary of δaδi analyses.
**Table S3:** Candidate regions and genes identified by the XP‐EHH test from *rehh* program, and functional annotation of the significant SNPs included in those regions.

## Data Availability

Raw sequencing data generated as part of this project are available under the Bioproject PRJNA1261573. NCBI accession numbers are also provided in Table S1. The SNP dataset provided as a VCF file and related documentation that support the findings of this study are available via the data. InDoRES DataVerse (CNRS—MNHN, France) at DOI: https://doi.org/10.48579/PRO/WXJJ52. Codes, notebooks, and related documentation that support the findings of this study are also available via github (https://github.com/jdaron/wilding). Data and code reuse is granted under a CC‐BY licence.
