# Supplementary material for: Genomic Signatures of Microgeographic Adaptation in Anopheles coluzzii Across Urban, Rural, and Forested Environments in Gabon
Source: Mol Ecol. 2026 Apr 20;35:e70349. doi: 10.1111/mec.70349 (PMC13093868; doi:10.1111/mec.70349)
Supplement: Supplementary file 1 — Figure S1: (A) Bioinformatic workflow from the reads cleaning, mapping to a the AgamP4 PEST reference genome, and SNP genotyping and quality filtering procedures used. (B) Schematic overview of the different analysis and the input dataset used. Figure S2: Sequencing coverage depth and SNP density along the genome. Figure S3: Kinship analysis across the Gabonese dataset estimated with pair‐wise IBD estimator (PI_HAT) between samples in PLINK (Anderson et al. 2010). Figure S4: Extended population structure analysis (A) Scree‐plot showing the variance fraction explained by each principal component of the PCA for the African An. coluzzii samples (combining the Gabonese and AG1000G datasets) represented in Figure 1. (B) PCA of the 77 An. coluzzii mosquitoes from Gabon retained for further analysis using biallelic unlinked SNPs from the euchromatic regions of the chromosome 3. The bar chart shows the percentage of variance explained by each principal component. Figure S5: Analysis of population structure and genetic ancestry in An. coluzzii considering the Gabonese populations in perspective with those from the Ag1000G. Figure S6: Pairwise population differentiations (F ST ) among populations of An. coluzzii. Figure S7: Test of departure from random mating expectation (panmixia) between pairs of populations performed using δaδi. Figure S8: δaδi model selection based on the AIC score obtained for 8 different models and 100 replicates per model. Figure S9: Population‐specific proportion and functional categories of SNPs with significant XP‐EHH values. Figure S10: Goodness‐of‐fit between empirical and simulated data under the 5 different types of selection scenarios of selective sweep in the diploS/HIC analysis. Figure S11: Graphical representation of the confusion matrix for each population obtained from the CNN classifier of diploS/HIC. [file MEC-35-e70349-s001.pdf]

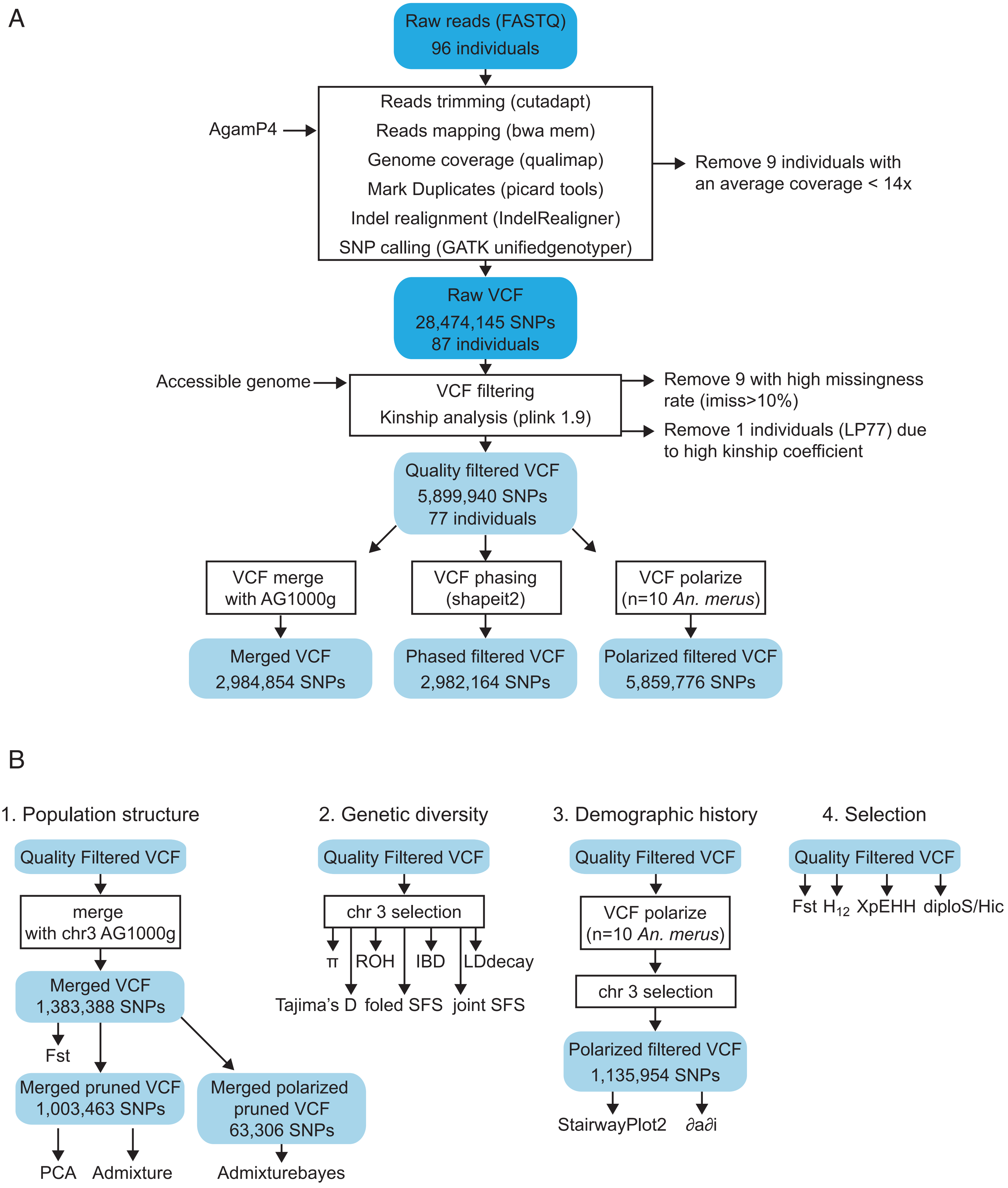

Figure S1: (A) Workflow of the reads mapping and SNP genotyping procedures used. (B) Schematic overview of the different analysis and the input dataset used.

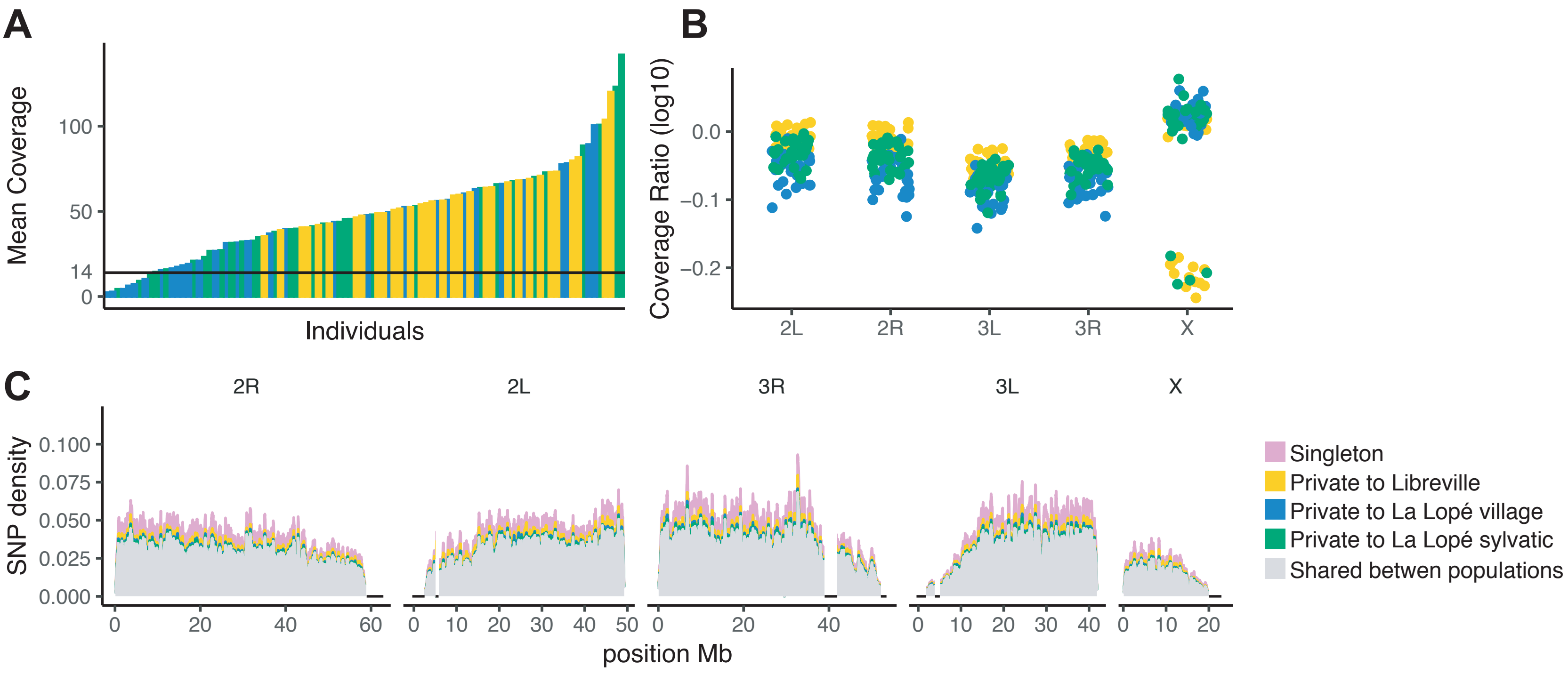

Figure S2: Sequencing coverage depth and SNP density along the genome. (**A**) Distribution of the mean sequencing depth of the 96 samples. Bars represent individual mosquito samples and are color-coded according to their sampling origin: Libreville (yellow); La Lopé village (blue); and La Lopé sylvatic (green). The horizontal line represents the coverage cut-off at 14x used to exclude samples below that threshold. (**B**) Coverage ratio of the mean sequencing depth for each chromosome over the coverage of the whole genome. This allowed to assign the sex of each individual, which was unknown for the larvae from Libreville (see Supplementary Table S1). (**C**) Density of the high-quality SNPs in 200-kb non-overlapping windows over the genome.

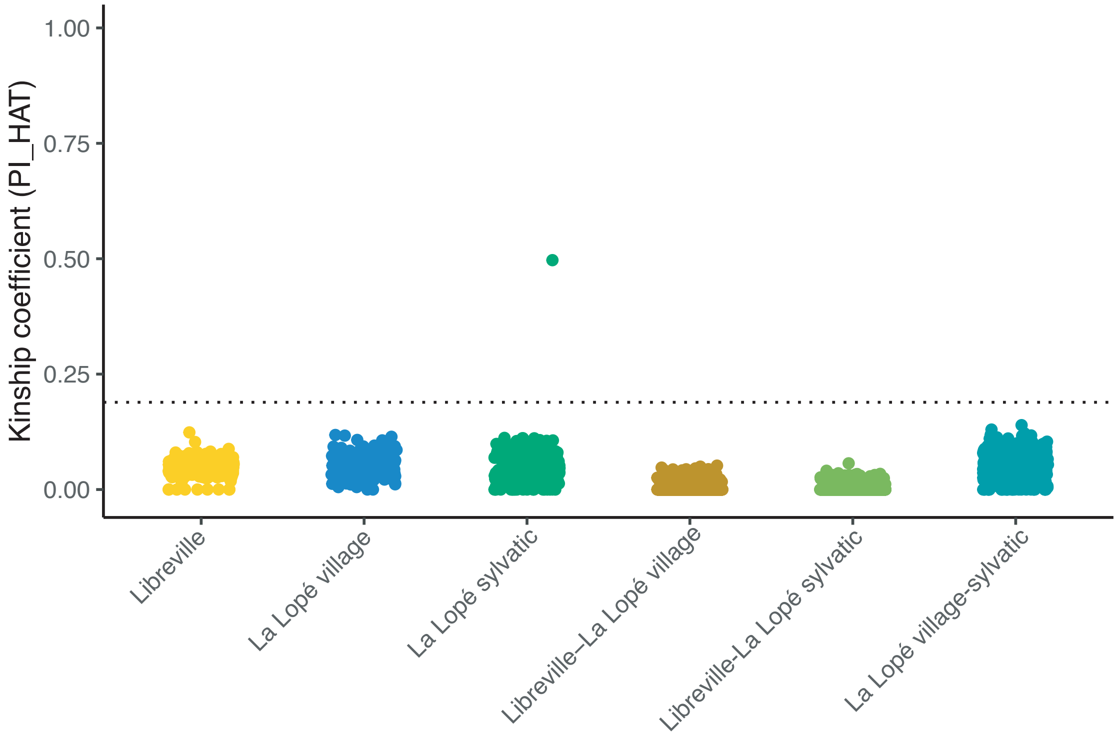

Figure S3: Kinship analysis across the Gabonese dataset estimated with pair-wise IBD estimator (PI_HAT) between samples in PLINK. The threshold 0.1875 represents the half-way point between 2nd and 3rd degree relatives and is a common cut-off to use (Anderson et al., 2010).

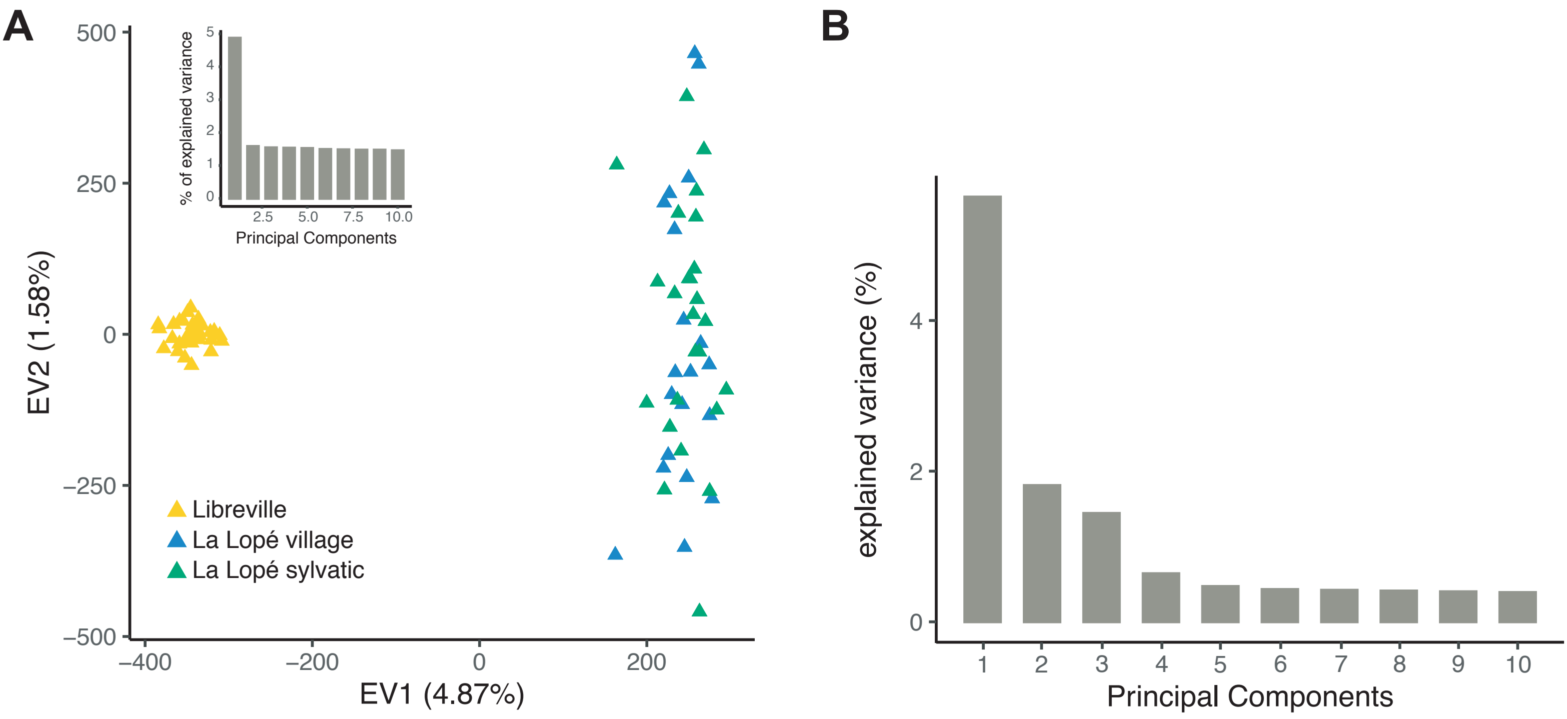

Figure S4: (**A**) PCA of the 77 *An. coluzzii* mosquitoes from Gabon retained for further analysis using biallelic SNPs from the euchromatic regions of the chromosome 3. The bar chart shows the percentage of variance explained by each principal component. (**B**) Scree-plot showing the variance fraction explained by each principal component of the PCA for the African *An. coluzzii* samples (combining the Gabonese and AG1000G datasets) represented in Figure 1.

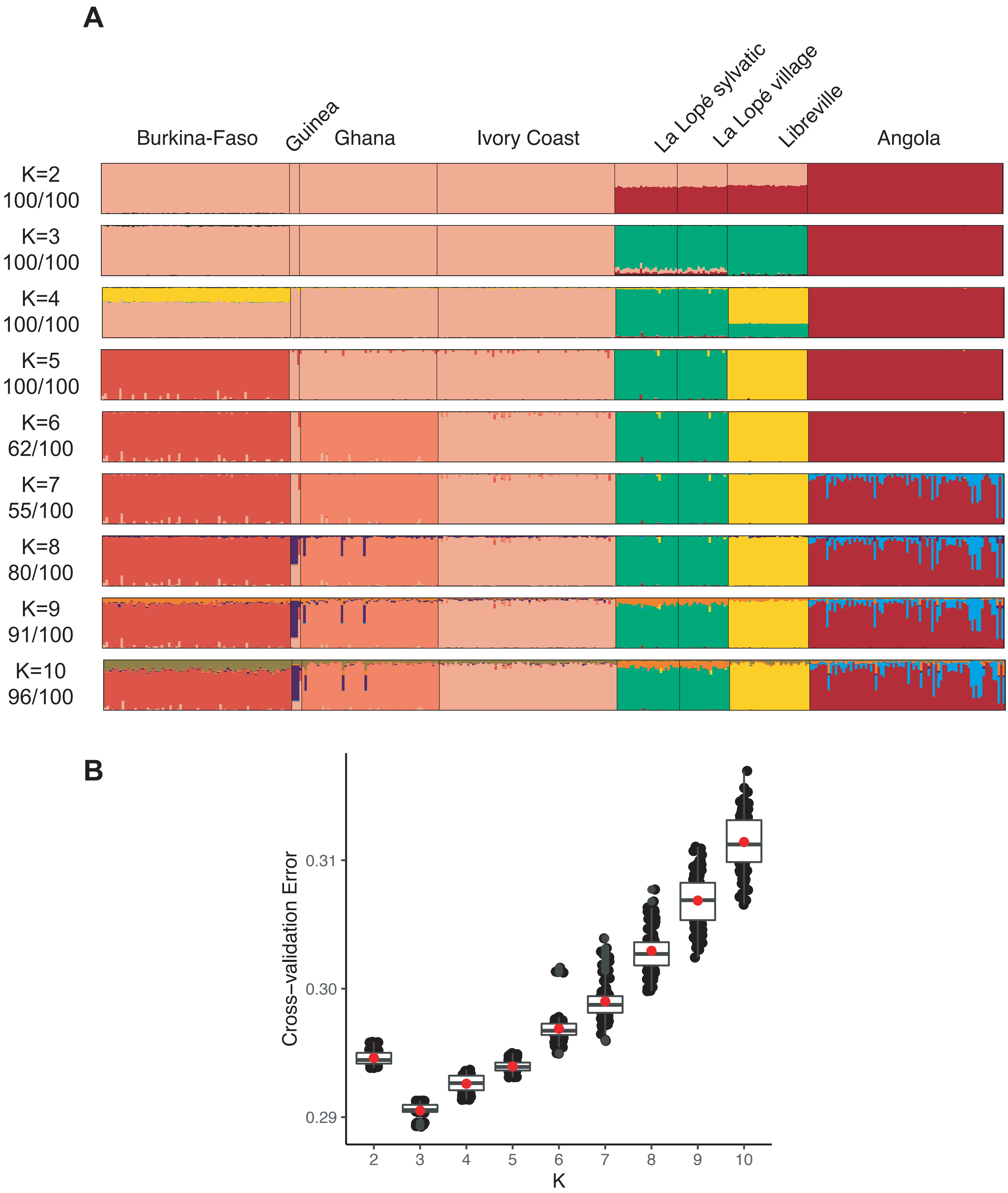

Figure S5: Analysis of population structure and genetic ancestry in *An. coluzzii* considering the Gabonese populations in perspective with those from the Ag1000G. (**A**) Individual ancestry proportions (from K=2 to K=10) were estimated using the ADMIXTURE program. Each vertical bar represents an individual mosquito grouped according to sampling location and colored according to the proportion of the genome inherited from each of the K ancestral clusters tested. (**B**) Box-plots showing the average (red dots), median, and interquartile values of the cross-validation (CV) error rate estimated using the ADMIXTURE program for each ancestral cluster tested (with K ranging between 2 and 10). Black dots show the CV error rate values for 100 replicated runs at each tested K values. K=3 was chosen as the best-fitted solution for our SNP dataset, since that value minimizes the CV error rate.

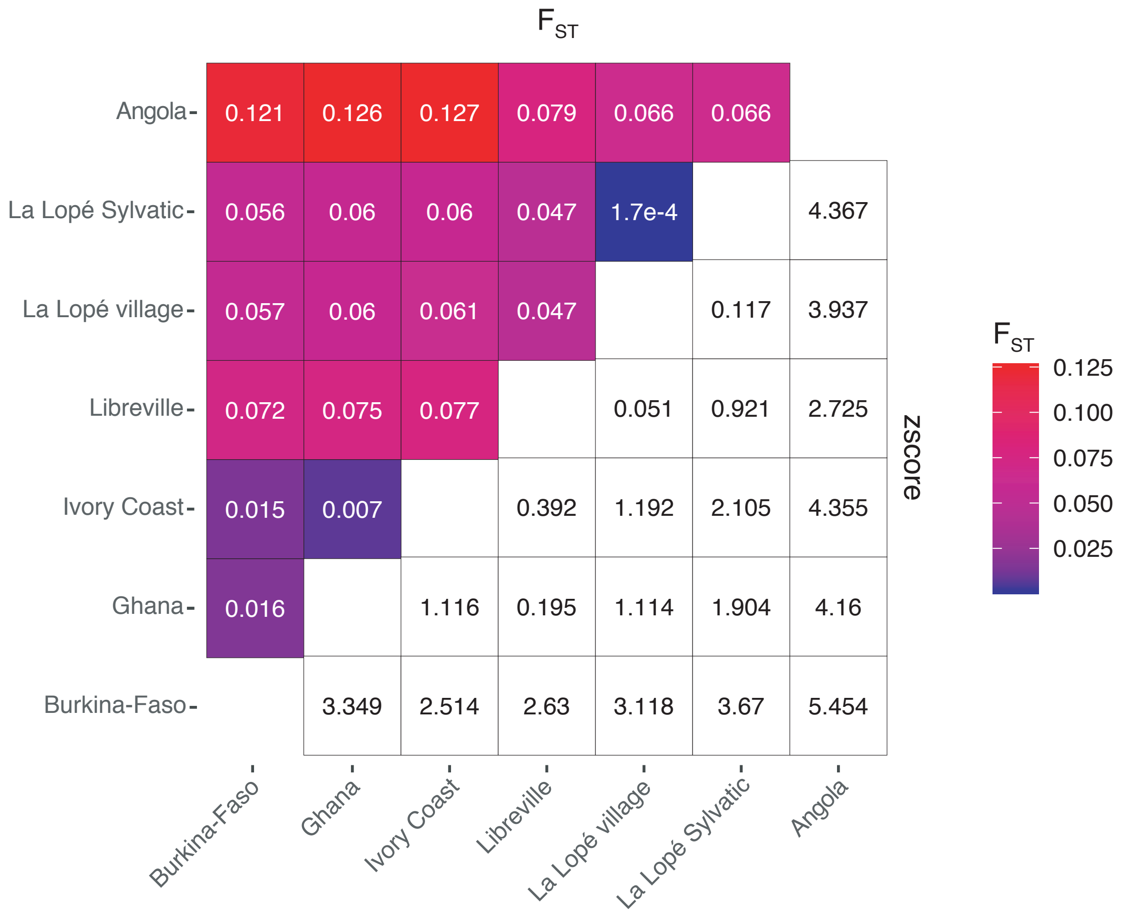

Figure S6: Pairwise population differentiations (F_{ST}) among populations of *An. coluzzii*. Average differentiation in allele frequency estimated using the F_{ST} statistics between pairs of populations. Upper left portion of the matrix shows average F_{ST} values between each population pair. Bottom right portion of the matrix shows the z-score for each F_{ST} value estimated via a block-jackknife procedure.

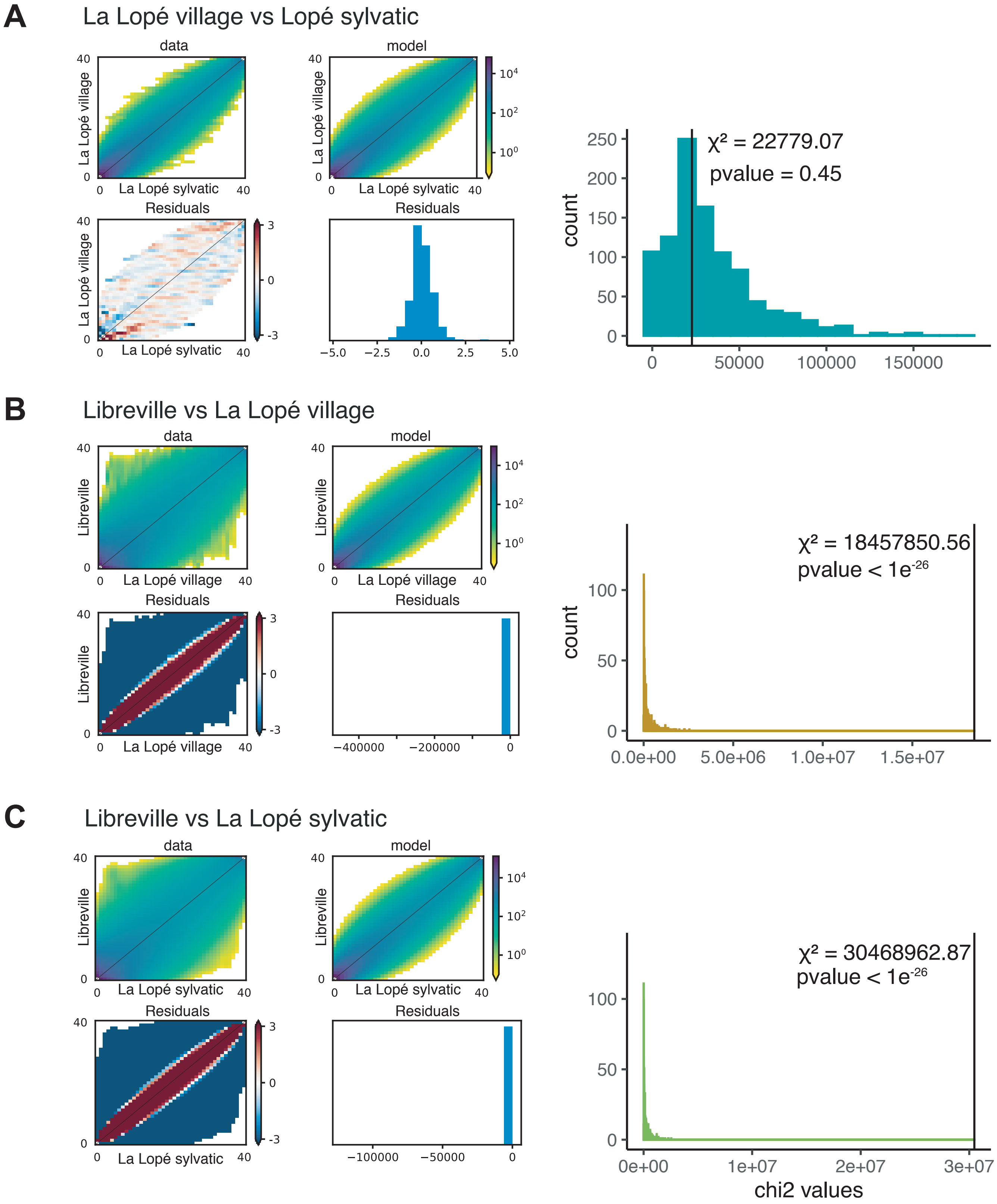

Figure S7: Test of departure from random mating expectation (panmixia) between pairs of populations performed using δaδi. The left panels represent the observed joint site frequency spectrum (jSFS) between populations pairs along with a model fit and residuals using δaδi, for a "scramble" model where individuals are permuted across population. On the right panel, the null distribution of χ2 values is obtained by measuring the deviation between 1000 replicates of permuted individual labels across the population pair to the scramble model. Vertical black line and value correspond to the χ2 value calculated between the observed jSFS and the scramble jSFSmodel. This test of departure from panmictic expectation was performed for all population pairs including : La Lope village versus La Lope sylvatic (**A**), Libreville versus La Lope village (**B**), and Libreville versus La Lope sylvatic (**C**).

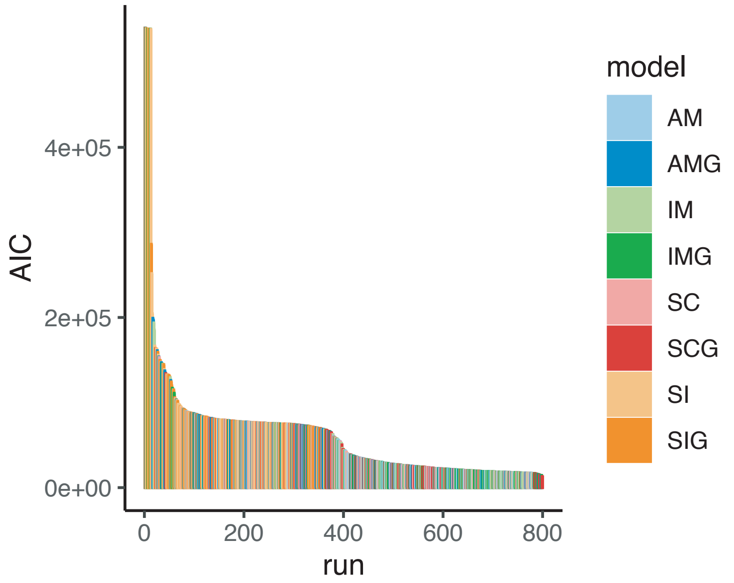

Figure S8: δaδi model selection based on the AIC score obtained for 8 different models with 100 replicates. The lowest AIC score was observed for the model secondary contact with growth (SCG).

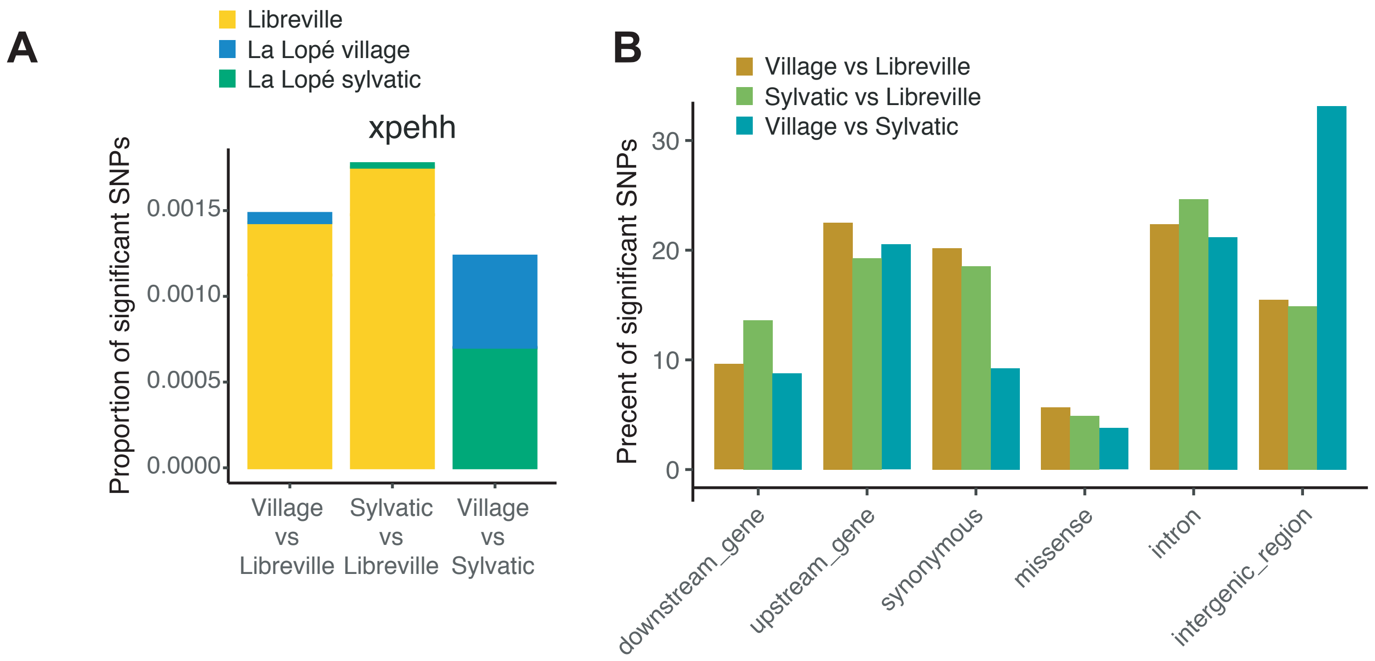

Figure S9: Population-specific proportion and functional categories of SNPs with significant XP-EHH signals. (**A**) Barplot representing the proportion of SNPs displaying significant p-value for the XP-EHH score for each of the 3 pairwise population comparisons. Color code represents the population in which the SNP has been found significant (Yellow – LBV; Green – LLP sylvatic; Blue – LLP village). (**B**) Functional annotation of the SNPs identified as significant using the XP-EHH scores in each of the three pairwise comparisons.

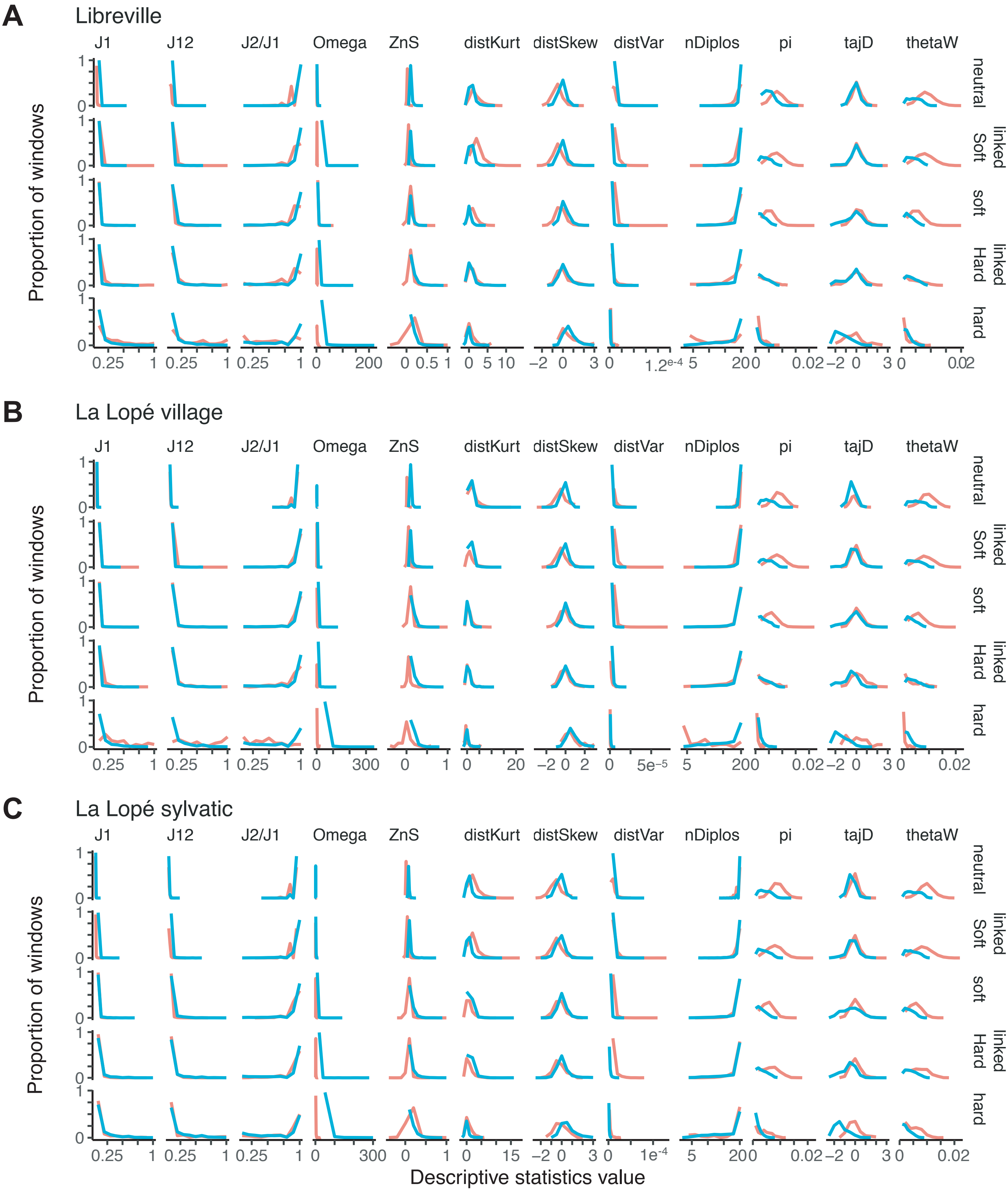

Figure S10: Goodness-of-fit between empirical and simulated data under the 5 different types of selection scenarios of selective sweep in the *diploS/HIC* analysis. The distributions obtained for each of the 12 summary statistics used in *diploS/HIC* and for each population (LBV, LPV, LPS) are displayed for the simulated (blue) and empirical (red) data.

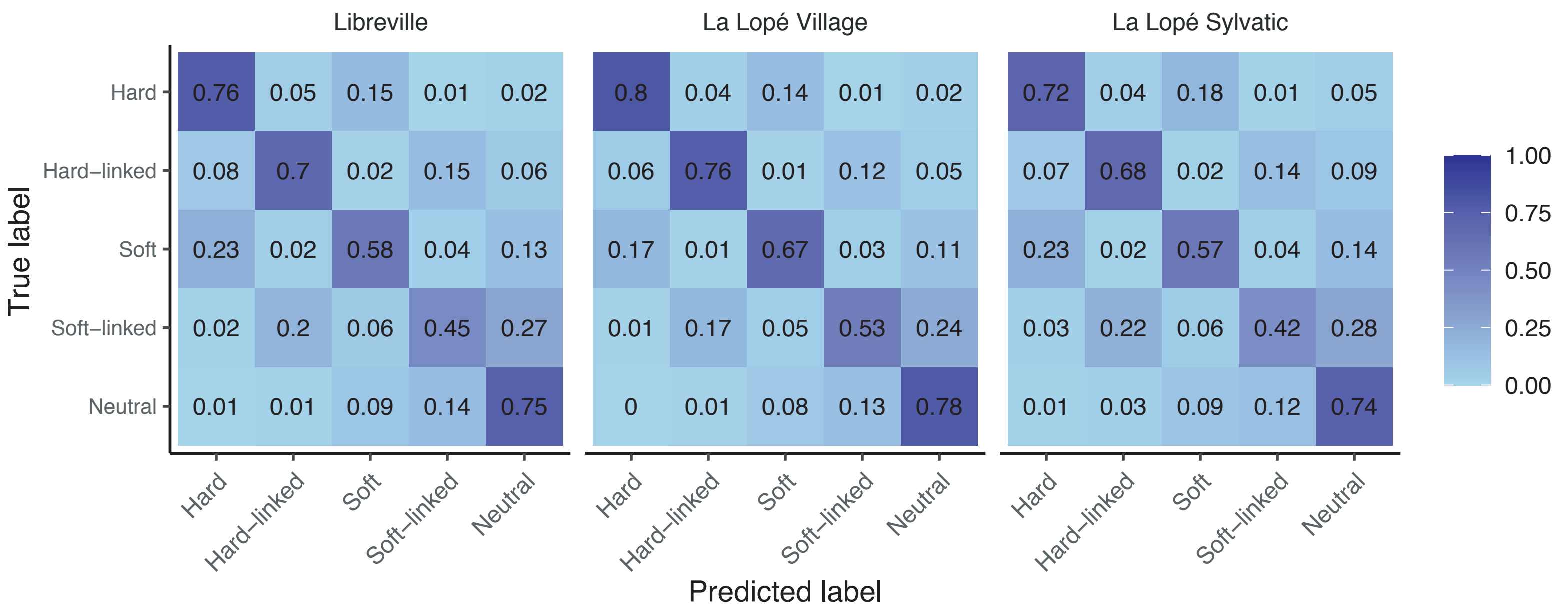

Figure S11: Graphical representation of the confusion matrix for each population. Predicted labels are displayed on the X-axis, and true labels are on the Y-axis. The values in the matrix represent the proportion of analysis windows assigned to each predicted category compared to their true categories. Diagonal values indicate the accuracy of each class, showing the proportion of correct predictions.
